# Exposing Racial Discrimination: Implicit & Explicit Measures–The *My Body, My Story* Study of 1005 US-Born Black & White Community Health Center Members

**DOI:** 10.1371/journal.pone.0027636

**Published:** 2011-11-18

**Authors:** Nancy Krieger, Pamela D. Waterman, Anna Kosheleva, Jarvis T. Chen, Dana R. Carney, Kevin W. Smith, Gary G. Bennett, David R. Williams, Elmer Freeman, Beverley Russell, Gisele Thornhill, Kristin Mikolowsky, Rachel Rifkin, Latrice Samuel

**Affiliations:** 1 Department of Society, Human Development and Health, Harvard School of Public Health, Boston, Massachusetts, United States of America; 2 Haas School of Business, University of California, Berkeley, California, United States of America; 3 RTI International, Waltham, Massachusetts, United States of America; 4 Psychology & Neuroscience and Duke Global Health Institute, Duke University, Durham, North Carolina, United States of America; 5 Departments of African and African American Studies and Sociology, Harvard University, Cambridge, Massachusetts, United States of America; 6 Center for Community Health Education Research & Service, Inc., Office of Urban Health Programs and Policy, Bouve College of Health Sciences, Northeastern University, Boston, Massachusetts, United States of America; 7 Boston, Massachusetts, United States of America; 8 Sharon, Massachusetts, United States of America; 9 Abt Associates, Cambridge, Massachusetts, United States of America; 10 Brigham & Women's Hospital, Boston, Massachusetts, United States of America; 11 Channing Laboratories, Boston, Massachusetts, United States of America; Royal Holloway, University of London, United Kingdom

## Abstract

**Background:**

To date, research on racial discrimination and health typically has employed explicit self-report measures, despite their potentially being affected by what people are able and willing to say. We accordingly employed an Implicit Association Test (IAT) for racial discrimination, first developed and used in two recent published studies, and measured associations of the explicit and implicit discrimination measures with each other, socioeconomic and psychosocial variables, and smoking.

**Methodology/Principal Findings:**

Among the 504 black and 501 white US-born participants, age 35–64, randomly recruited in 2008–2010 from 4 community health centers in Boston, MA, black participants were over 1.5 times more likely (p<0.05) to be worse off economically (e.g., for poverty and low education) and have higher social desirability scores (43.8 vs. 28.2); their explicit discrimination exposure was also 2.5 to 3.7 times higher (p<0.05) depending on the measure used, with over 60% reporting exposure in 3 or more domains and within the last year. Higher IAT scores for target vs. perpetrator of discrimination occurred for the black versus white participants: for “black person vs. white person”: 0.26 vs. 0.13; and for “me vs. them”: 0.24 vs. 0.19. In both groups, only low non-significant correlations existed between the implicit and explicit discrimination measures; social desirability was significantly associated with the explicit but not implicit measures. Although neither the explicit nor implicit discrimination measures were associated with odds of being a current smoker, the excess risk for black participants (controlling for age and gender) rose in models that also controlled for the racial discrimination and psychosocial variables; additional control for socioeconomic position sharply reduced and rendered the association null.

**Conclusions:**

Implicit and explicit measures of racial discrimination are not equivalent and both warrant use in research on racial discrimination and health, along with data on socioeconomic position and social desirability.

## Introduction

One important challenge confronted by empirical research on racial discrimination and health is how best to measure the relevant exposures at the relevant levels – whether structural, institutional, interpersonal, or internalized [1]–[3]. To date, most studies in this still relatively new field of inquiry have focused on testing associations between people's self-reported experiences of racial discrimination and their concurrent or subsequent health status [1]–[6]. A unique value of these observational data, as opposed to data obtained via experimental methods, is their promise for providing information on the population distribution of the exposure and its contribution to racial/ethnic inequalities in health [1], [2].

Yet, as long-recognized for any type of self-report data [7], important concerns pertain to the validity of what people are able and willing to self-report and how this may be influenced by the methods employed (e.g., types of questions asked, whether by interviewers or by self-administered questionnaires) [1]–[6]. Beyond the standard caveats, self-report data on racial discrimination have the distinction of plausibly being affected by the very exposure under study, given the power differentials and social sensitivities involved. At issue is what people are willing and/or able to self-report, that is, identify as experiences due to racial discrimination, and how this is linked to their social position, including extent of subjective and objective disempowerment versus entitlement [1], [2], [8], [9].

One concern, reflecting dominant views that alleged victims are too quick to claim they are targets of discrimination and benefit from doing so [10]–[12], tends to contrast “perceived” versus externally-defined “real” discrimination, with the distinction typically hinging on the motivations of the alleged perpetrator. Of note, use of the term “perceived discrimination,” with this meaning implied, if not outright stated, is widespread in the scientific literature [1]–[6], as is the conflation of “perceived” with “self-reported” (implying that all “perceived” discrimination is in fact self-reported) [1], [8], [9]. A second set of concerns focuses on a different array of issues involving three other aspects of perception. The first pertains to social desirability and safety: people may acknowledge to themselves they have been a target of discrimination, but – given the well-known phenomenon of social desirability, referring to situations in which people report answers they think will be deemed “right” or “acceptable,” whether or not these answers truly reflect their own views [7], [13] – they are unwilling to disclose this information because it feels rude, transgressive, or unsafe to do so [1], [2], [5], [10], [12]. The second involves internalized oppression: people's judgment may be affected by imposed powerlessness, with discriminatory treatment deemed deserved and due to their own inadequacies [1]–[5], [8], [9]. The third concerns frame of reference: especially germane to immigrants, people's ability to identify racial discrimination requires familiarity with a society's racial/ethnic conventions and classifications [14]–[17].

In the past few years, two new approaches for addressing the complexities of measuring self-report data on experiences of racial discrimination have emerged in the public health literature. The first, to our knowledge thus far used by only 14 studies [9], [17]–[29], is to include and control for measures of social desirability. The second, to date employed in only two studies [8], [9], involves use of the now well-validated implicit association test (IAT), a computer-based timed reaction measure designed to study phenomena for which self-report data might not fully capture what people think and feel [30]–[32]. Its development spurred in part by the challenge of measuring racial prejudice at a time when explicit endorsement of racially biased views has become increasingly unacceptable [11], [12], the IAT measures the strength of associations between concepts [30]–[32]. The underlying presumption, supported by neuroscience and social cognitive research, is that the stronger the mental association between two concepts, the shorter the time needed to classify them as “belonging to the same category” in a sorting task (with the speed of the test reducing possibilities for conscious cognitive correction [30]–[33]). In the case of racial prejudice, for example, the reaction-time contrast is between how long it takes to link the constructs of “white” versus “black” respectively with, say, “good” versus “bad” [30].

The first two investigations to use an IAT for racial discrimination measured associations between participants' sense of both themselves and their racial/ethnic group as being either a target versus perpetrator of discrimination [8], [9]. The first of these studies was conducted using a small community-based sample [8], the second employing larger pool of web-based and highly educated participants [9]. Both studies found, as predicted, weak associations between the implicit and explicit measures of discrimination, akin to the low correlations observed in other studies that have examined implicit versus explicit measures of phenomena likely subject to self-presentational bias [32], [33], and together provided suggestive evidence of their joint saliency for analyzing health outcomes.

We accordingly designed the *My Body, My Story* study both to build on and address the limitations of our prior two investigations, so as to improve research methods for investigating the impact of racial discrimination on health. Guided by the ecosocial theory of disease distribution and its focus on how people literally biologically embody their societal and ecologic context, at multiple levels, across the lifecourse and historical generations [34]–[36], our research project seeks to triangulate diverse types of evidence relevant to assessing the health impact of racial discrimination: people's self-report, the IAT, and data obtained by physical measurement. In this first paper, we describe our study protocol and the study participants and test our *a priori* hypotheses that: (1) observed associations between the implicit and explicit measures of racial discrimination would be weak; (2) associations with other psychosocial covariates potentially affected by concerns pertaining to social desirability, and also those signifying more power (e.g., higher income or education), would be stronger for the explicit measures, and weaker or non-existent for the implicit measures; and (3) both the implicit and explicit measures would be relevant for analysis of health outcomes and social inequalities in health, with the chosen example – current smoking – selected because in major review articles it is one of the few non-psychological health outcomes reported to demonstrate reasonably consistent positive associations with exposure to racial discrimination [1], [3]–[6].

## Methods

The *My Body, My Story* study is a cross-sectional epidemiologic investigation based on a random sample drawn from the rosters of four community health centers in Boston, MA (USA). We chose to recruit participants from community health centers because they not only serve populations that are low income, medically underserved, and diverse in their racial/ethnic composition [37], but also are trusted community-based organizations, which is especially important for recruiting participants from social groups that have been subjected to social and economic deprivation and historically exposed to unethical research practices [38]. The study protocol was approved by the Harvard School of Public Health Office of Human Research Administration (protocol #11950-127), which additionally covered 3 of the 4 health centers (through reciprocal IRB agreements), and it was separately approved by the fourth community health center's Institutional Review Board. All participants provided written informed consent. The study was funded by the National Institutes of Health/National Institute on Aging (1 R01 AG027122-01), and the funder had no role in study design, data collection and analysis, decision to publish, or preparation of the manuscript.

### Study population: eligibility, recruitment, and enrollment

**Figure 1:**
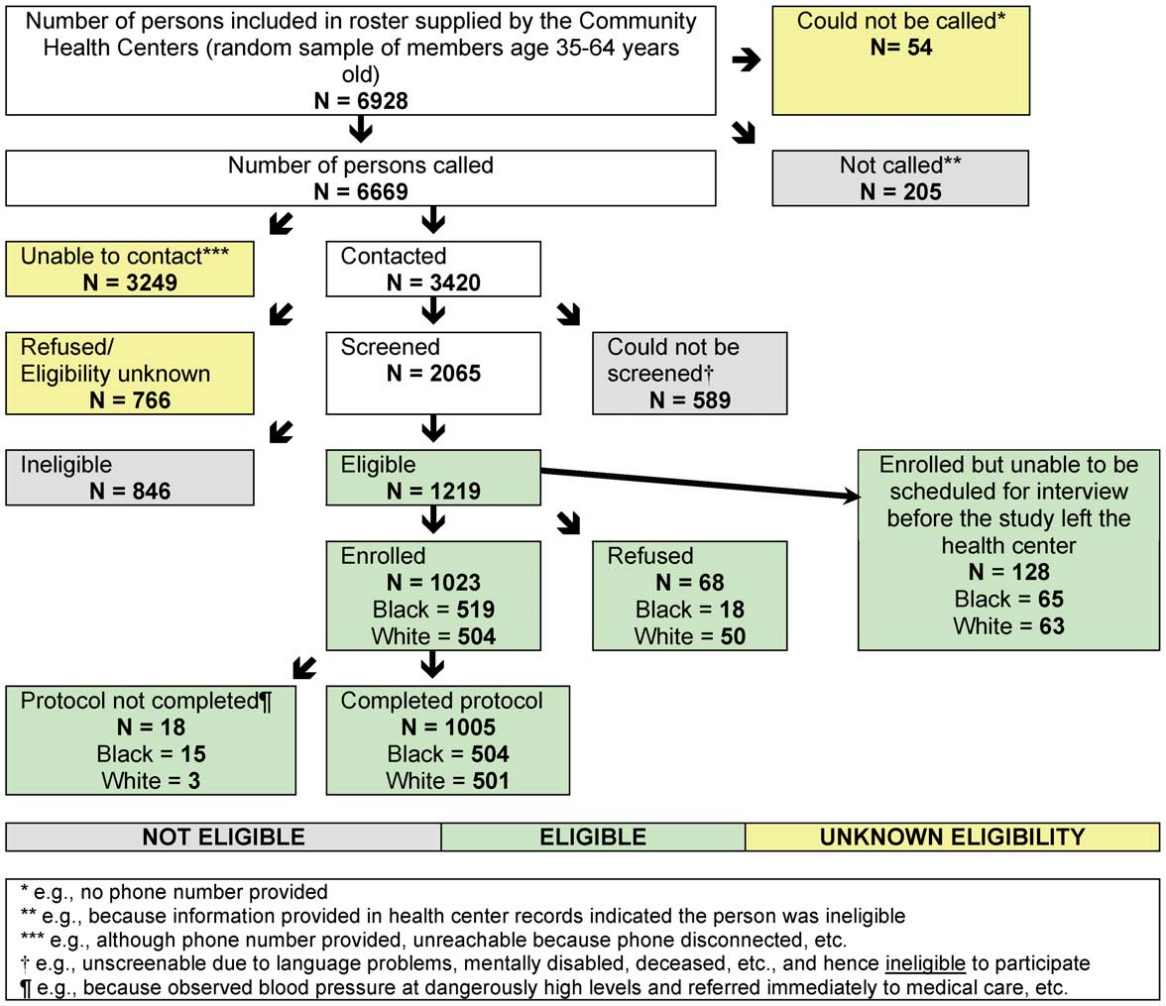
Study enrollment: *My Body My Story* (Boston, MA, 2008–2010).

Recruitment commenced in August 2008 (about a year after the US economy entered what has been termed the “Great Recession,” following the housing market collapse in 2007–2008, and shortly before the bank failures in the fall of 2008 [39]) and ceased in December 2010, when we reached the study target of enrolling, with completed protocols, 500 US-born self-identified white non-Hispanic participants and 500 US-born self-identified black non-Hispanic participants, all English-speaking and between 35 and 64 years old. The study sample size of 500 per group was based on power analyses to ensure we would have at least 80% power to detect, within each group, hypothesized effect sizes of 0.276 or more for high versus low exposure to racial discrimination for: blood pressure, waist circumference, cholesterol level, glucose level, and Framingham risk score, along with hypothesized prevalence differences for smoking and for the metabolic syndrome. Figure 1 provides the detailed flow chart describing whom we were and were not able to recruit.

In brief, each community health center generated a roster of members age 35 to 64 from their membership, who were randomly assigned to batches of approximately 250 to 300 people. The health centers then sent each batch, successively, a letter, prepared at the 6^th^ grade literacy level, inviting them to participate; each mailing to a new batch was sent once we were close to exhausting recruitment from the prior batch. In the letter, we informed members that the study was to “help understand how life experiences affect our health” and stated that if they were chosen to participate, they would be given a $75 grocery card to thank them for their time. Also provided was an opt-out number (on a phone line accessible only to health center staff) that the members could call, within 7 days of receiving the letter, if they did not want to be contacted; according to the community health centers, only 30 persons (<0.4% of members sent letters) chose to opt-out of being contacted. Our rationale for the opt-out, versus opt-in, approach was to minimize problems arising from selection bias [40]. After the deadline for opting-out passed, the community health centers then forwarded to our study team the names and telephone number(s) of health center members who had not opted-out (N = 6928). The study research assistants then attempted to reach these members by phone, with calls alternating between morning and evening on weekdays and weekends, up to a maximum of 10 contact attempts. The purpose of the call was to screen for eligibility and, if the person was eligible and agreed to participate, to schedule an appointment.

To be deemed eligible, a potential participant had to self-identify as white or black, be US-born, speak English, be age 35 to 64, and be cognitively able to provide information on eligibility and for ethical written informed consent. Our rationale for these criteria were that: (1) self-report is the preferred method for obtaining data on the social construct of race/ethnicity, including for research on racial discrimination and health [1]–[6]; (2) to date, much of the US research on racial discrimination and its contribution on racial/ethnic health disparities has focused on US non-Hispanic black vs white comparisons [1]–[6], such that results could readily be compared to the published literature; (3) requiring participants to be US-born and English-speaking would avoid incommensurate data due either to different frames of reference or differences in questionnaire meaning due to translation, both issues demonstrated to affect estimates of self-reported racial discrimination among US immigrants [14]–[17]; (4) the selected age range (35 to 64 years old) permitted participants to accumulate the relevant exposures potentially leading to chronic disease while simultaneously reducing the likelihood of age-by-pathology selection bias, a form of self-selection bias affecting inclusion of sicker participants [41]; and (5) ethical written informed consent can only be provided by persons who are mentally competent [42].

Persons who agreed to enroll were then sent a follow-up packet with: (a) information about the time and place of their appointment (an exam room at their health center, unless they chose to be interviewed at a designated examination room at the Harvard School of Public Health), and (b) the study's written consent form and, for one health center, the HIPAA “HHSI Notice of Privacy Practices” and the “HHSI Acknowledgement of Receipt of Notice of Privacy Practices” form. Also included were instructions not to eat or drink anything (except water and required medications) after 8 pm the night before their study appointment. The day prior to their appointment, the potential participants received a reminder call from a study research assistant regarding: the time and place of appointment; the need to bring in the signed and completed informed consent form (and HIPAA forms for the one health center); the instructions about not eating or drinking prior to the appointment; a reminder to bring their eyeglasses, if needed; and a request to bring in the labeled containers for any prescribed medications they currently were taking.

As summarized in Figure 1, among the 6928 community health center members we attempted to screen, of the 3420 persons contacted, 1219 met the study inclusion criteria, of whom fully 94.4% agreed to participate in the study (black: 97.0%; white: 91.9%). Among the 1023 persons enrolled, 1005 (504 black and 501 white) completed the study protocol; among the remaining 18, following our protocol stipulations, we discontinued 10 (9 black, 1 white) and triaged them to referral for medical assistance at the health center, since we found they had dangerously high blood pressure, and 8 (6 black, 2 white) were unable to complete the study survey instrument. Considering only potential participants determined to be eligible, the study response rate accordingly was 82.4% (black: 86.0%, white: 81.4%), per the American Association of Public Opinion Research (AAOPR) definition of response rate as “(completed interviews)/eligible” [43].

### Study protocol: components and counterbalancing

Upon arrival for the study appointment, the potential participant was met by a study research assistant, who verbally reviewed the written consent form (and also, for the one health center, the HIPAA “Acknowledgement of Receipt of Notice of Privacy Practices” form) with the participant, and the person was enrolled only if s/he signed and submitted the form(s). If the potential participant was a no-show, study staff attempted to reschedule the appointment; after the fifth missed appointment, the final disposition for this person was categorized as “refused” to enroll.

The study protocol, designed to take 75 minutes to complete, included four components: (1) the survey instrument, self-administered on a laptop computer via the Audio-Computer Assisted Self-Interviewing (ACASI) methodology [44]; (2) the IAT, also self-administered on the laptop; (3) the physical exam; and (4) the fingerprick, for on-site analysis of specified biomarkers detected in the obtained bloodspots. We used the ACASI methodology because it is a technique that improves the likelihood of obtaining sensitive information and enables persons with low literacy to respond [44], whereby questions shown on the screen are also read out-loud, over a headphone, via the digitally-recorded audio component, and participants respond by pressing the indicated keys on a masked keyboard. In our study population, 25% of participants used the headphones the entire time. To avoid order effects, the order of the ACASI and IAT components of the protocol were counterbalanced, as were the order of the different explicit discrimination questions included in the ACASI survey.

At the end of the protocol, the participant was debriefed by the study research assistant and given both the $75 grocery card and a 26-page resource booklet (also prepared at the 6^th^ grade literacy level, so as to be accessible to all participants). This booklet included: (1) a two-page debriefing statement about the study; (2) information on each participant's blood pressure, body measurements (standing and sitting height, waist circumference, tibia length, body mass index), and cholesterol (total, LDL, HDL), glucose, and triglyceride levels, along with information to help interpret their levels and provide guidance on keeping these levels healthy; and (3) a resource list for government agencies and other organizations providing legal assistance to address racial discrimination, plus a list of local organizations providing mental health and social services.

### Study measures

#### Sociodemographic

Because racial discrimination may affect health both independently of – and in interaction with – socioeconomic position across the lifecourse and at different levels [1]–[5], [36], [45]–[47] we used previously validated questions to obtain data on: childhood and adult social class, household income, household poverty (defined in relation to the US poverty thresholds corresponding to the year in which the interview was conducted [48], [49], and taking into account the number and age of persons supported by the household income), public assistance, housing tenure, debt, wealth, and educational level (for the participants, their household, and their parents/guardians)[18], [50]–[52]. To characterize the socioeconomic composition of the participants' neighborhood, we additionally used ArcGIS [53] to geocode each participant's residential street address to the census tract, which we then linked to the 2005–2009 American Community Survey data on census tract poverty level [54], [55]. Data on the participants' age and state of birth were used to determine what we refer to as their Jim Crow birthplace status, referring to states that did versus did not legally permit racial discrimination [56] prior to the 1964 passage of the US Civil Rights Act that rendered such discrimination illegal and the 1965 Voting Rights Act that abolished literacy tests aimed at preventing black and poor white citizens from voting [57], [58].

#### Racial discrimination: explicit

The two main explicit self-report measures of exposure to racial discrimination that we employed were: (1) the Experiences of Discrimination (EOD) instrument [18], [59], [60], and (2) the Everyday Discrimination Scale (EDS): short form [61]. Both are psychometrically validated [18] and are among the most commonly employed self-report measures of racial discrimination used in health research [1], [3], [4], [6].

The EOD is a 9-item measure that is conceptualized as measuring “self-reported experiences of discrimination,” recognizing that the data obtained depend on people's willingness and/or ability to report these experiences [1], [18], [36], as per the predicted weak association we found between the EOD and the IAT [8], [9]. Building on a prior instrument developed by Krieger [59], [60], the EOD asks participants if they have ever experienced discrimination due to race, color, or ethnicity in 9 specified domains (at school; getting hired or getting a job; at work; getting housing; getting medical care; getting service in a store or restaurant; getting credit, bank loans, or a mortgage; on the street or in a public setting; from the police or in the courts), and if so, the frequency of such experiences (once, 2–3 times, 4 or more times). Two additional questions pertain to response to unfair treatment (“accept it as a fact of life” vs “try to do something about it,” “talk to other people about it” vs “keep it to yourself”) [18], [59], [60]. On the basis of prior research [1], [17]–[21], we defined exposure categories as no exposure (0 situations), moderate exposure (1 or 2 situations), and high exposure (3 or more situations).

The short version of the Everyday Discrimination scale (EDS) [61]–[63] is a self-report measure that asks if participants have experienced unfair treatment, and if so, what they think is the main reason these experiences happened to them. For each of the 5 situations listed (pertaining to being treated with less courtesy, receiving poorer service, people acting as if you are not smart, people acting as if they are afraid of you, and being threatened or harassed), the questions ask about frequency of occurrence (ranging from “almost every day” to “less than once a year”). Among the 9 attribution options, two are directly relevant to racial discrimination (“your race”; “your ancestry or national origin”); the others pertain to gender, sexual orientation, age, height, weight, and “some other aspect of your physical appearance.” Because research indicates that estimates of exposure differ for self-report of (a) unfair treatment (without attribution), compared to (b) unfair treatment attributed to race/ethnicity [25], [64], [65], we analyzed both sets of responses (respectively referred to as “EDS (any)” and “EDS (race)”).

Additionally, to gauge participants' recent and lifetime appraisal of racial discrimination directed at not only themselves but also their racial/ethnic group, we included four questions used in previous studies [9], [18]. These questions pertained to how much participants worried about racial discrimination as a child and in the past year, personally and for their racial/ethnic group.

#### Racial discrimination: implicit

We employed the two IATs used in our prior studies, which involved cognitive tasks using reaction-time methodology to measure the strength of the participants' mental association between themselves and their racial/ethnic group as a target versus perpetrator of discrimination [8], [9]. The specifics of the IAT methodology are well-described in the social psychology literature [30]–[33], [66], and programming resources to develop IATs are available on-line [67].

In order to get participants used to the proper speed of executing the test, they first performed a training using the standard “insect-flower” pairing test [8], [9], [66]. This training test contrasts the time it takes to make associations between the words (a) “flower” and “good,” and (b) “bugs” with “bad,” and then compares what happens when participants alternatively are asked to pair (c) “flower” with “bad” and (d) “bugs” with “good.” A difference in average matching speed for opposite pairings determines the IAT score, a measure of strength of association. Participants are typically aware that they are making these connections but unable to control them given the rapid response times and structure of the test.

**Figure 2:**
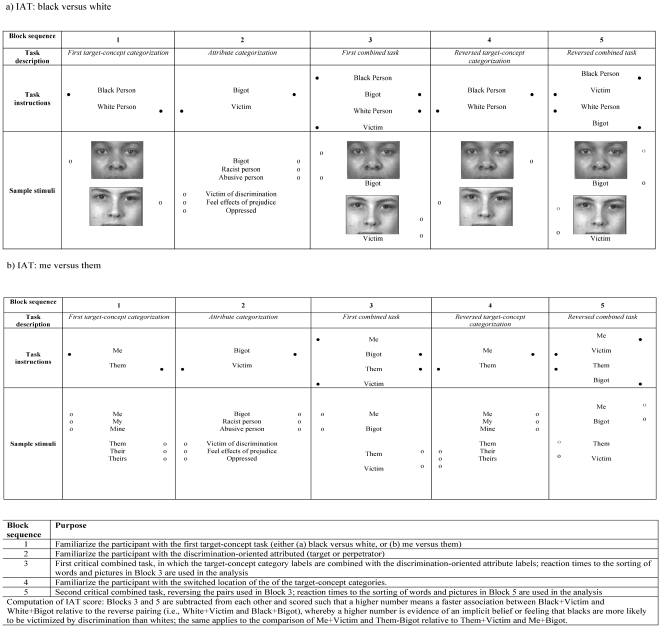
Implicit association test (IAT) for associations with target versus perpetrator of discrimination for: (a) black versus white, and (b) me versus them.

After completing the training test, participants were then administered the two IATs in randomly determined sequences, so as to minimize (and also allow us to model) order effects. These IATs were introduced by anchoring language explicitly addressing whether the participant had been a “target of discriminatory behavior.” As shown in Figure 2, the IAT employed: (a) two sets of targets: (1) “self” and (2) “group”, and (b) two sets of attribute categorization terms: (1) “abuser,” “racist,” and “bigot”; and (2) “target,” “victim,” and “oppressed.” These attribute terms were selected based on pilot studies we conducted with Boston community-based participants, including members of the study community health centers [8], [9]. The IAT's core contrast concerned how quickly or not participants linked words or images that pertain to self or to their group to words or images (photographs of persons who are black or white) that pertain to being a victim or perpetrator of racial discrimination. The difference in speed (in milliseconds) for the two associations produced the raw IAT score, which is then normalized following standard IAT protocol [66], [67]. A score of 0 indicates a participant equally felt s/he was a victim and bigot, whereas a high score indicates the participant felt s/he was more a victim than a bigot, and a low negative score indicates s/he felt s/he was more of a bigot than a victim. Because preliminary inspection of the data showed that, despite the counterbalancing, some order effects were still apparent (as if often the case [66]), we centered the scores on the value of the IAT sequence “white/black/them/me” and controlled for IAT order in the analytic models.

#### Psychosocial

To test hypotheses regarding the association of social desirability with the different measures of racial discrimination, we used the validated RAND 5-item social desirability scale [68], previously employed in six studies on racial discrimination and health [9], [17]–[21]. Two additional measures, documented to be associated with self-reported racial discrimination and potentially act as confounders or effect modifiers of its relationship to health status [1], [3]–[6], [69] were: (a) hostility, measured using the validated 8-item New-Buss Hostility Scale [70], [71], and (b) racial/ethnic centrality, measured using the validated 8-item Centrality subscale of the Multidimensional Inventory of Black Identity [72], which we also employed to measure white identity.

#### Smoking

We employed questions about smoking behavior drawn from the 2000 US National Health Interview Survey Sample Adult Core Questionnaire [73]. Following standard practice, we defined “current smokers” to be individuals who both reported smoking more than 100 cigarettes in their lifetime and smoked on all or some days during the previous month [73].

### Statistical analyses

To understand the properties of the data set, we first assessed the distribution of each variable, including extent of missingness, overall and stratified by race/ethnicity, and then additionally stratified by gender and by socioeconomic position. Next, to guide our modeling of variables in the multivariable analyses, we visually inspected bivariate plots of associations between the 3 explicit and 2 implicit measures of discrimination both with each other and the additional study variables and performed appropriate chi-square, trend, Spearman correlation, and t-tests as warranted. Observing no departures from linearity, we then ran analytic logistic regression models for the models with current smoking as the outcome. To address the modest level of missingness (typically under 5%, except for the socioeconomic variables), we implemented multiple imputation via the Amelia II program [74] to create 10 imputed data sets, with the imputation model including all variables employed in the smoking models, and then combined estimates across the imputed data sets using standard methods. All analyses were conducted in SAS [75].

## Results

Tables 1 and 2 provide data on the 1005 US-born *My Body, My Story* study participants (504 black, 501 white), whose mean age was slightly below 50. Reflecting the typical socioeconomic and gender composition of urban community health center members [76], 75.5% of participants (black: 84.6%; white: 66.4%) had completed high school but not college; among the 90% whose poverty level could be determined, 27.4% were below the US poverty line (black: 33.8%; white: 21.3%); and women predominated (black: 69.2%; white: 63.1%). Most participants had been born in Massachusetts (black: 60.3%; white: 72.5%) and over 90% of their parents/guardians were US-born. A far higher proportion of the black compared to white participants, however, had been born in a Jim Crow state prior to 1965 (27.2% vs 3.8%). Fully 43.8% of the black participants and 34.5% of the white participants currently smoked cigarettes.

**Table 1:** Study participant characteristics: sociodemographic and socioeconomic profile: 504 black US-born and 501 white US-born community health center members, *My Body My Story* (Boston, MA, 2008–2010).

Variable		Observed data		Missing: N (%)	
		Black	White	Black	White
Age (mean (SD)):	years	48.6 (8.0)	49.0 (8.0)	0	0
Gender (%):	women	**69.2**	**63.1**	0	0
Household income: categorical (%):	<$48,000 per year (%)	**58.8**	**46.4**	62 (12.3)	35 (7.0)
Poverty: (% US poverty line) (%):	<50% below	**11.1**	**6.9**	63 (12.5)	36 (7.2)
	50% to 100%	**22.7**	**14.4**		
	>100 and <200%	**21.8**	**18.5**		
	> = 200 and <400%	**19.1**	**19.1**		
	> = 400%	**25.4**	**41.1**		
Education (%):	less than high school	**16.1**	**9.9**	0	4 (0.8)
	> = high school but <4 yrs college	**68.5**	**56.5**		
	> = 4 yrs college	**15.5**	**33.6**		
Occupational class (%):	Owner/self-employed/supervisor	**20.9**	**34.6**	1 (0.2)	4 (0.8)
	Non-supervisory employee	**36.2**	**29.0**		
	Unemployed/Not in paid labor force/Other	**42.9**	**36.4**		
Debt (%):	owe > = $5,000 to creditors	**39.8**	**53.3**	142 (28.2)	66 (13.2)
Wealth (other than home) (%):	no financial assets*	**78.7**	**54.2**	72 (14.3)	40 (8.0)
	any financial assets	**21.3**	**45.8**		
	high financial assets (> = $5,000)	**7.2**	**30.6**	88 (17.5)	63 (12.6)
Household received public assistance (%):	as a child	**52.8**	**33.2**	50 (9.9)	25 (5.0)
	in the last year	**43.6**	**28.6**	18 (3.6)	8 (1.6)
Housing tenure (%):	rent for cash	**68.2**	**46.6**	26 (5.2)	12 (2.4)
	paying mortgage	**22.6**	**42.5**		
	paying mortgage	**3.8**	**6.1**		
	occupy without paying rent cash	**5.4**	**4.7**		
Census tract poverty (2005–2009) (%):	<5% below poverty	**6.8**	**18.5**	18 (3.6)	36 (7.2)
	5–9% below poverty	**8.4**	**27.1**		
	10–19% below poverty	**32.1**	**30.5**		
	20–39% below poverty (“poverty area”)	**38.5**	**20.6**		
	> = 40% below poverty (“extreme poverty area”)	**14.2**	**3.2**		
Jim Crow birthplace status§ (%):	born in Jim Crow state before 1965	**27.2**	**3.8**	0	1 (0.2)
	born in Jim Crow state during or after 1965	**3.0**	**2.0**		
	not born in Jim Crow state	**69.8**	**94.2**		
Parent/guardian born in US (%):	mother/female guardian	**94.8**	**91.2**	1 (0.2)	2 (0.4)
	father/male guardian	93.7	90.5	11 (2.2)	7 (1.4)
Parents'/guardians' education: highest attained by either parent/guardian (%):	less than high school	**27.3**	**12.0**	83 (16.5)	33 (6.7)
	> = high school but <4 yrs college	**55.1**	**51.3**		
	> = 4 yrs college	**17.6**	**36.8**		
Parents'/guardians' education: at most high school degree or GED (general equivalence diploma (%):	mother/female guardian	**70.5**	**61.3**	90 (17.9)	39 (7.8)
	father/male guardian	**72.0**	**54.9**	136 (27.0)	64 (12.8)

Note: **values in bold** indicate that, for the specified variables, the difference in distribution by race/ethnicity is statistically significant (p<0.05), using relevant non-parametric tests.

*financial assets: bonds, treasury notes, IRA's, certificates of deposit, shares of stocks or mutual funds; does not include value of home.

§ Jim Crow states/district : District of Columbia plus Alabama, Arizona, Arkansas, Delaware, Florida, Georgia, Kansas, Kentucky, Louisiana, Maryland, Mississippi, Missouri, New Mexico, North Carolina, Oklahoma, South Carolina, Tennessee, Texas, Virginia, West Virginia, and Wyoming [51].

**Table 2:** Study participant characteristics: distributions of implicit and explicit racial discrimination measures, psychosocial variables, and smoking: 504 black US-born and 501 white US-born community health center members, *My Body My Story* (Boston, MA, 2008–2010).

Variable		Observed data		Missing: N (%)	
		Black	White	Black	White
**Racial discrimination**					
***Explici*** *t*					
Racial discrimination (Experiences of Discrimination [EOD]):				2 (0.4)	1 (0.2)
continuous (range: 0–9) (mean (SD))		**3.8 (2.7)**	**1.2 (1.7)**		
categorical (%):	0 situations	**14.1**	**50.2**		
	1–2 situations	**21.7**	**32.2**		
	3+ situations	**64.1**	**17.6**		
Everyday discrimination (EDS):				9 (1.8)	4 (0.8)
EDS (any): continuous (range: 0–5) (mean (SD))		**2.5 (1.6)**	**2.2 (1.5)**		
EDS (any): categorical (%):		86.3	85.1		
EDS (race)*: continuous for unfair treatment due to race (range: 0–5) (mean (SD))		**1.8 (1.8)**	**0.5 (1.3)**		
EDS (race)*: categorical (%):	report unfair treatment (>1 x/yr) due to race	**59.2**	**18.5**		
Worried about racial discrimination (%):	as a child, against self	**70.2**	**20.2**	1 (0.2)	2 (0.4)
	as a child, against own racial/ethnic group	**69.8**	**30.1**	1 (0.2)	2 (0.4)
	in last year, against self	**64.0**	**20.8**	1 (0.2)	2 (0.4)
	in last year, against own racial/ethnic group	**71.8**	**31.5**	1 (0.2)	2 (0.4)
***Implicit***					
IAT effect (mean, SD, p-value):	Total Black vs White (de-trended and centered on w/b/t/m)	**0.26 (0.32)** ‡	**0.13 (0.39)** ‡	10 (2.0)	10 (2.0)
	Total Me vs Them (de-trended and centered on w/b/t/m)	**0.24 (0.36)** ‡	**0.19 (0.34)** ‡	11 (2.2)	10 (2.0)
**Psychosocial measures**					
Response to unfair treatment (%):	take action and talk to others (act/talk)	68.2	64.3	1 (0.2)	0
	take action and keep to self (act/quiet)	9.3	10.0		
	accept as fact of life and talk to others (accept/talk)	14.7	16.4		
	accept as fact of life and keep to self (accept/quiet)	7.8	9.4		
Racial/ethnic centrality (range: 1–5) (mean (SD))		**3.3 (0.7)**	**2.5 (0.7)**	1 (0.2)	4 (1.0)
Social desirability: continuous (range: 0–100) (mean (SD))		**43.8 (31.7)**	**28.2 (29.5)**	27 (5.4)	11 (2.2)
Hostility: continuous (range: 8–40) (mean (SD))		18.9 (6.3)	18.4 (6.1)	27 (5.4)	11 (2.2)
**Smoking (%):**	current smoker	**43.8**	**34.5**	0	0
	ex-smoker	**16.9**	**34.5**		
	never smoker	**39.3**	**30.9**		

Note: **values in bold** indicate that, for the specified variables, the difference in distribution by race/ethnicity is statistically significant (p<0.05), using relevant non-parametric tests.

*“race” includes, as specified reasons, “race” and “ancestry or national origin”; all non-racial exposures scored as 0.

‡ IAT effect (within racial/ethnic group) statistically significant (p<0.001).

Despite the commonality of being community health center members, however, the black participants' current and lifetime socioeconomic profile was notably worse than that of the white participants (Table 1), with all differences statistically significant (p<0.05) unless otherwise noted. Specifically, they were over 1.5 times more likely to be impoverished (33.8% vs 21.3%), to live in a census tract meeting the federal definition of poverty area (> = 20% of persons below poverty) (52.6% vs. 23.9%), to rent where they lived (68.2% vs 46.6%), to have received public assistance in the last year (43.6% vs 28.6%) as well as when a child (52.8% vs 33.2%), to have not graduated from high school (16.1% vs 9.9%), and to have parents/guardians who had not graduated from high school. Conversely, the white participants were over 1.6 times more likely to have incomes that placed them at 4 times the poverty level (41.1% vs 25.4%), to live in a census tract in which <5% of the population was impoverished (18.5% vs. 6.8%), to be paying a mortgage for their home (42.5% vs 22.6%), to have at least $5,000 in assets (not including their home) (30.6% vs 7.2%), to have graduated from college (33.6% vs 15.5%), and to have parents/guardians who also graduated from college (36.8% vs 17.6%). Only for debt (owing at least $5000 to creditors, which presumes having access to credit) did the white participants fare worse than their black counterparts (53.3% vs 39.8%).

Table 2 additionally provides data on the distribution of the explicit and implicit racial discrimination measures and other psychosocial covariates, with all differences statistically significant (p<0.05) unless stated otherwise. In Tables 3–6, these results are further stratified by gender and by two different socioeconomic measures: poverty level and educational level.

**Table 3:** Distribution, by poverty level and gender, of the implicit and explicit measures of racial discrimination: 504 black US-born and 501 white US-born participants, *My Body My Story* (Boston, MA, 2008–2010) (observed data).

		Poverty level: <200% vs > = 200% US poverty level							
		Black				White			
		Women		Men		Women		Men	
		<200%(n = 178)	> = 200%(n = 136)	<200%(n = 67)	> = 200%(n = 60)	<200% (n = 124)	> = 200%(n = 172)	<200% (n = 61)	> = 200%(n = 108)
**Racial discrimination**									
***Explicit***									
Racial discrimination (Experiences of Discrimination [EOD]):									
continuous (range: 0–9) (mean (SD))		3.5 (2.5)	3.3 (2.5)	5.2 (2.5)	5.2 (2.8)	1.1 (1.6)	1.0 (1.6)	1.9 (2.3)	1.1 (1.5)
categorical (%):	0 situations	14.7	14.7	9.0	6.7	49.2	56.1	**39.3**	**50.9**
	1–2 situations	14.7	27.9	9.0	13.3	35.5	29.8	**31.2**	**33.3**
	3+ situations	61.0	57.4	82.1	80.0	15.3	14.0	**29.5**	**15.7**
Everyday discrimination (EDS):									
EDS (any): continuous (range: 0–5) (mean (SD))		2.6 (1.5)	2.4 (1.5)	3.0 (1.6)	2.6 (1.7)	1.8 (1.5)	2.1 (1.4)	2.7 (1.6)	2.2 (1.6)
EDS (any): categorical (%):	report unfair treatment (>1x/yr)	87.9	85.1	91.0	84.8	80.3	86.6	91.8	81.5
EDS (race) ‡: continuous for unfair treatment due to race (range: 0–5) (mean (SD))		1.7 (1.8)	1.8 (1.7)	2.5 (2.0)	2.1 (1.9)	0.4 (1.1)	0.4 (1.0)	0.9 (1.8)	0.6 (1.3)
EDS (race) ‡: categorical (%):	report unfair treatment (>1 x/yr) due to race	54.6	61.9	68.7	64.4	18.0	14.0	22.0	22.2
Worried about racial discrimination (%):	as a child, against self	62.4	66.7	88.6	83.3	18.7	15.8	**42.6**	**17.6**
	as a child, against own racial/ethnic group	65.2	64.7	83.6	81.7	30.1	26.9	41.0	27.8
	in last year, against self	62.4	59.6	73.1	71.7	21.1	17.0	**32.8**	**18.5**
	in last year, against own racial/ethnic group	69.7	68.4	82.1	76.7	28.5	28.7	**50.8**	**24.1**
***Implicit***									
IAT effect (mean, SD, p-value):	Total Black vs White (de-trended and centered on w/b/t/m)	0.22 (0.33)***	0.28 (0.32)***)	0.35 (0.29)***	0.30 (0.35)***	0.05 (0.33)	0.13 (0.40)***	−0.03 (0.39)	0.28 (0.36)***
	Total Me vs Them (de-trended and centered on w/b/t/m)	0.23 (0.36)***	0.23 (0.35)***	0.27 (0.36)***	0.34 (0.36)***	0.18 (0.29)***	0.24 (0.32)***	0.18 (0.39)**	0.11 (0.33)**

Note: **values in bold** indicate that, within the specified racial/ethnic-gender group, the distribution is **significantly different (p<0.05)** across socioeconomic strata, as based on 2-sided t-test for continuous variables and chi-square test for categorical variables; data presented: observed data (not including missing values).

‡ “race” includes, as specified reasons, “race” and “ancestry or national origin”; all non-racial exposures scored as 0.

For statistical significance of IAT effect (within racial/ethnic-gender-socioeconomic group): * = 0.01<p<0.05; ** = 0.001<p<0.01; *** = p<0.001.

**Table 4 pone-0027636-t004:** Distribution, by poverty level and gender, of the psychosocial variables and Jim Crow birthplace status: 504 black US-born and 501 white US-born participants, *My Body My Story* (Boston, MA, 2008–2010) (observed data).

		Poverty level: <200% vs > = 200% US poverty level							
		Black				White			
		Women		Men		Women		Men	
		<200%(n = 178)	> = 200%(n = 136)	<200%(n = 67)	> = 200%(n = 60)	<200% (n = 124)	> = 200%(n = 172)	<200% (n = 61)	> = 200%(n = 108)
**Psychosocial measures**									
Response to unfair treatment (%):	take action and talk to others (act/talk)	70.2	71.3	74.6	78.3	67.7	70.4	49.2	63.0
	take action and keep to self (act/quiet)	8.4	5.2	10.5	5.0	5.7	7.6	19.7	11.1
	accept as fact of life and talk to others (accept/talk)	13.5	20.5	9.0	11.7	19.4	15.7	16.4	13.9
	accept as fact of life and keep to self (accept/quiet)	7.9	2.9	6.0	5.0	7.3	6.4	14.8	12.0
Racial/ethnic centrality (range: 1–5) (mean (SD))		3.2 (0.6)	3.3 (0.7)	3.3 (0.6)	3.4 (0.6)	2.4 (0.6)	2.5 (0.7)	2.5 (0.7)	2.4 (0.7)
Social desirability: continuous (range: 0–100) (mean (SD))		44.8 (31.0)	46.8 (32.5)	41.0 (30.8)	38.9 (30.2)	**38.4 (32.0)**	**28.5 (28.9)**	19.0 (24.6)	21.9 (26.7)
Hostility: continuous (range: 8–40) (mean (SD))		19.1 (6.4)	18.1 (6.6)	19.5 (5.0)	19.1 (5.5)	18.1 (5.9)	17.0 (6.1)	**21.6 (5.4)**	**18.2 (6.0)**
**Jim Crow birthplace status** § **(%):**	born in Jim Crow state before 1965	23.6	23.5	37.3	30.0	3.2	4.7	1.6	3.7
	born in Jim Crow state during or after 1965	2.3	3.7	1.5	6.7	0.0	1.2	1.6	5.6
	not born in Jim Crow state	74.2	72.8	61.2	63.3	96.8	94.2	99.7	90.7

Note: **values in bold** indicate that, within the specified racial/ethnic-gender group, the distribution is **significantly different (p<0.05)** across socioeconomic strata, as based on 2-sided t-test for continuous variables and chi-square test for categorical variables; data presented: observed data (not including missing values).

§ Jim Crow states/district : District of Columbia plus Alabama, Arizona, Arkansas, Delaware, Florida, Georgia, Kansas, Kentucky, Louisiana, Maryland, Mississippi, Missouri, New Mexico, North Carolina, Oklahoma, South Carolina, Tennessee, Texas, Virginia, West Virginia, and Wyoming [51].

**Table 5 pone-0027636-t005:** Distribution, by education and gender, of the implicit and explicit measures of racial discrimination: 504 black US-born and 501 white US-born participants, *My Body My Story* (Boston, MA, 2008–2010) (observed data).

		Education level: <4 yrs college vs > = 4 years college							
		Black				White			
		Women		Men		Women		Men	
		<4 yrs college(n = 292)	> = 4 yrs college(n = 57)	<4 yrs college(n = 134)	> = 4 yrs college(n = 21)	<4 yrs college(n = 203)	> = 4 yrs college(n = 109)	<4 yrs college(n = 127)	> = 4 yrs college(n = 58)
**Racial discrimination**									
***Explicit***									
Racial discrimination (Experiences of Discrimination [EOD]):									
continuous (range: 0–9) (mean (SD))		**3.1 (2.5)**	**4.1 (2.7)**	**5.0 (2.7)**	**6.4 (1.5)**	**1.2 (1.7)**	**0.8 (1.5)**	1.6 (2.1)	1.1 (1.3)
categorical (%):	0 situations	17.9	12.3	9.0	0.0	**48.3**	**60.2**	47.2	46.6
	1–2 situations	27.2	19.3	13.4	4.8	**33.0**	**31.5**	29.1	37.9
	3+ situations	54.8	68.4	77.6	95.2	**18.7**	**8.3**	23.6	15.5
Everyday discrimination (EDS):									
EDS (any): continuous (range: 0–5) (mean (SD))		2.4 (1.5)	2.3 (1.6)	2.8 (1.7)	3.3 (1.5)	2.1 (1.4)	1.9 (1.5)	**2.6 (1.7)**	**2.0 (1.4)**
EDS (any): categorical (%):	report unfair treatment (>1x/yr)	87.0	80.4	85.7	95.2	87.0	80.7	85.8	84.5
EDS (race) ‡: continuous for unfair treatment due to race (range: 0–5) (mean (SD))		**1.6 (1.7)**	**2.1 (1.7)**	2.1 (2.0)	2.9 (1.9)	0.5 (1.1)	0.3 (1.1)	**0.6 (1.5)**	**0.9 (1.4)**
EDS (race) ‡: categorical (%):	report unfair treatment (>1 x/yr) due to race	**54.4**	**69.6**	62.7	81.0	**19.0**	**10.1**	**16.5**	**36.2**
Worried about racial discrimination (%):	as a child, against self	61.9	71.9	85.1	85.7	**19.7**	**9.4**	29.9	20.7
	as a child, against own racial/ethnic group	62.2	75.4	81.3	85.7	**34.0**	**17.8**	34.7	29.3
	in last year, against self	58.1	66.7	73.1	81.0	**24.6**	**9.4**	26.8	15.5
	in last year, against own racial/ethnic group	**65.0**	**80.7**	81.3	81.0	**34.0**	**22.4**	32.3	36.2
***Implicit***									
IAT effect (mean, SD, p-value):	Total Black vs White (de-trended and centered on w/b/t/m)	0.22 (0.32)***	0.30 (0.36)***	0.32 (0.31)***	0.40 (0.31)***	−0.01 (0.36)	0.33 (0.37)***	0.10 (0.40)**	0.35 (0.32)***
	Total Me vs Them (de-trended and centered on w/b/t/m)	0.23 (0.36)***	0.18 (0.34)***	0.31 (0.35)***	0.34 (0.39)***	0.20 (0.34)***	0.26 (0.26)***	0.10 (0.34)**	0.18 (0.37)***

Note: **values in bold** indicate that, within the specified racial/ethnic-gender group, the distribution is **significantly different (p<0.05)** across socioeconomic strata, as based on 2-sided t-test for continuous variables and chi-square test for categorical variables; data presented: observed data (not including missing values).

‡ “race” includes, as specified reasons, “race” and “ancestry or national origin”; all non-racial exposures scored as 0.

For statistical significance of IAT effect (within racial/ethnic-gender-socioeconomic group): * = 0.01<p<0.05; ** = 0.001<p<0.01; *** = p<0.001.

**Table 6:** Distribution, by education and gender, of psychosocial variables, and Jim Crow birthplace status: 504 black US-born and 501 white US-born participants, *My Body My Story* (Boston, MA, 2008–2010) (observed data).

		Education level: <4 yrs college vs > = 4 years college							
		Black				White			
		Women		Men		Women		Men	
		<4 yrs college(n = 292)	> = 4 yrs college(n = 57)	<4 yrs college(n = 134)	> = 4 yrs college(n = 21)	<4 yrs college(n = 203)	> = 4 yrs college(n = 109)	<4 yrs college(n = 127)	> = 4 yrs college(n = 58)
**Psychosocial measures**									
Response to unfair treatment (%):	take action and talk to others (act/talk)	66.3	79.0	67.9	66.7	65.0	75.2	55.9	62.1
	take action and keep to self (act/quiet)	8.3	5.3	13.4	9.5	8.4	3.7	15.0	12.1
	accept as fact of life and talk to others (accept/talk)	17.2	12.3	9.7	19.1	18.7	15.6	13.4	17.2
	accept as fact of life and keep to self (accept/quiet)	8.3	3.5	9.0	4.8	7.9	5.5	15.8	8.6
Racial/ethnic centrality (range: 1–5) (mean (SD))		**3.2 (0.7)**	**3.5 (0.6)**	**3.3 (0.6)**	**3.7 (0.6)**	2.5 (0.6)	2.4 (0.7)	2.5 (0.7)	2.5 (0.6)
Social desirability: continuous (range: 0–100) (mean (SD))		46.4 (31.2)	44.2 (31.0)	39.2 (31.0)	34.0 (26.8)	**35.5 (32.6)**	**26.2 (25.8)**	**24.5 (28.3)**	**14.0 (19.0)**
Hostility: continuous (range: 8–40) (mean (SD))		18.7 (6.5)	17.8 (7.0)	19.7 (5.5)	20.7 (5.7)	**18.6 (6.2)**	**15.9 (5.5)**	**20.3 (5.7)**	**18.3 (6.5)**
**Jim Crow birthplace status** § **(%):**	born in Jim Crow state before 1965	25.3	21.0	35.1	19.1	3.5	5.5	**1.6**	**6.9**
	born in Jim Crow state during or after 1965	2.7	3.5	3.0	4.8	0.5	1.8	**0.0**	**12.1**
	not born in Jim Crow state	71.9	75.4	61.9	76.2	96.1	92.7	**98.4**	**81.0**

Note: **values in bold** indicate that, within the specified racial/ethnic-gender group, the distribution is **significantly different (p<0.05)** across socioeconomic strata, as based on 2-sided t-test for continuous variables and chi-square test for categorical variables; data presented: observed data (not including missing values).

§ Jim Crow states/district : District of Columbia plus Alabama, Arizona, Arkansas, Delaware, Florida, Georgia, Kansas, Kentucky, Louisiana, Maryland, Mississippi, Missouri, New Mexico, North Carolina, Oklahoma, South Carolina, Tennessee, Texas, Virginia, West Virginia, and Wyoming [51].

As expected, the black compared to white participants reported the most exposure to racial discrimination (Table 2), with their self-reported levels anywhere from 2.5 to 3.7 times higher than those for the white participants: for both the EOD and EDS (race), both a higher mean score (3.2 vs 1.2, and 1.8 vs 0.5, respectively) and 64.1% vs 17.6% for 3 or more situations (EOD), with 59.2% vs 18.5% reporting unfair treatment in the past year due to race (EDS (race)). The effect size for the black vs. white comparisons of these two explicit measures was also very large: 1.01 for the EOD and 0.77 for the EDS (race). The difference in worry about racial discrimination against self and group when a child and in the last year was also evident, with self-reports ranging between 60 to 70% among the black participants, compared to 20 to 30% among the white participants.

By contrast, the black and white participants were almost equally likely to report unfair treatment without any attribution (EDS (any)). Although the continuous score for EDS (any) for the black participants was slightly higher (2.5 vs. 2.2; effect size for the black vs. white comparison = 0.23), the percent who reported such unfair treatment in the last year was equal (86.3% vs 85.1%) and these frequencies exceeded those reported for unfair treatment due to race (black: 1.5 times higher; white: 4.6 times higher).

Consonant with the explicit measures of racial discrimination, but not unfair treatment (unattributed), the IAT effect for black vs white as a target of discrimination was two times higher among the black compared to white participants (0.26 vs 0.13), with this difference demonstrating a moderate effect size of 0.36. The black/white difference for IAT for me vs them as a target of discrimination, while smaller, was likewise statistically significant (0.24 vs 0.19), but the effect estimate for the black vs. white difference was low, equaling only 0.15.

With regard to the additional psychosocial variables, the black and white participants reported similar responses to unfair treatment, with 68.2% and 64.3% stating they took action and talked to others, and only 7.8% and 9.4% stating they accepted such treatment as a fact of life and kept it to themselves. They likewise were similar in their mean scores for hostility (18.9 and 18.4). They notably differed, however, in their mean scores for social desirability, which were over 1.5 times higher in the black compared to white population (43.8 vs 28.2). Racial/ethnic centrality was also higher among the black compared to white participants (3.3 vs 2.5).

As shown in Tables 3–6, among the black and among the white participants, gender and socioeconomic differences (p<0.05) were evident for the explicit discrimination measures and the additional psychosocial variables (social desirability, hostility, racial/ethnic pride). No such differences, however, occurred for the implicit measures of discrimination. The socioeconomic differences, moreover, were more apparent in analyses stratified by education (dichotomized at <4 years vs > = 4 years college; Tables 5–6), which provided more extreme contrasts (lower percent in the high category) as compared to stratification by poverty level (dichotomized at <200% vs > = 200% poverty; Tables 3–4). Considering both sets of results together, the data indicate that among both the black women and men, explicit self-reports of racial discrimination were higher among those with more versus less socioeconomic resources, and also that at each socioeconomic level, reports were higher among men compared to women. By contrast, among the white participants, especially among women, explicit reports of racial discrimination, and also hostility, were higher among those with fewer socioeconomic resources. Social desirability scores were uniformly high among the black participants (between 35 to 45) and did not significantly vary by gender or socioeconomic position. Among the white population, however, social desirability scores were highest among the white women with the fewest economic resources (between 35 and 38, i.e., on par with the black participants) and were 2 to 3 times lower among the white men with the most economic resources (between 14 and 21).

Table 7 in turn presents the distribution of domains of discrimination, as measured by the EOD, simultaneously stratified by race/ethnicity and gender. Among the black participants, men were significantly more likely than women to self-report having experienced racial discrimination in all of the domains except one (“getting medical care”), with their odds ranging from 1.39 (95% CI 1.14, 1.70) for “on the street or in a public setting” to 2.29 (95% CI 1.87, 2.82) for “from the police or in the courts.” By contrast, among the white participants, there were no significant gender differences in the self-reports of racial discrimination, except for “from police or in the courts” (odds ratio for men compared to women: 1.62 (95% CI 1.87, 2.82)). Consequently, the magnitude of the odds ratios, comparing black to white participants, for self-reported experiences of racial discrimination were generally higher among men (ranging between 2 and 4) as compared to women (ranging between 1.6 and 3), and statistically significant interactions between race/ethnicity and gender (p<0.05) were evident for 4 of the 9 situations (“at school,” “getting a job,” “at work,” and “from the police and in the courts”).

**Table 7:** Domains of self-reported experiences of racial discrimination: distribution and comparisons by race/ethnicity and gender: 504 black US-born and 501 white US-born participants, *My Body My Story* (Boston, MA, 2008–2010) (observed data).

	Frequency (%)				Comparisons by gender and by race/ethnicity				Significance of interaction of race/ethnicity and gender
	Black		White		Odds Ratio (95% CI)				
EOD situation	Women	Men	Women	Men	Gender		Race/ethnicity		
					Black men vs Black women	White men vs White women	Black men vs White men	Black women vs White women	
At school	40.2	62.6	20.3	17.8	**1.58 (1.30, 1.92)**	0.92 (0.73, 1.16)	**2.78 (2.16, 3.56)**	**1.62 (1.37, 1.93)**	p<0.001
Getting a job	36.5	69.7	15.6	21.1	**2.00 (1.63, 2.45)**	1.20 (0.95, 1.52)	**2.93 (2.29, 3.75)**	**1.77 (1.46, 2.13)**	p = 0.001
At work	46.3	69.7	15.2	19.5	**1.63 (1.34, 2.00)**	1.16 (0.91, 1.47)	**3.08 (2.40, 3.96)**	**2.19 (1.82, 2.64)**	p = 0.031
Getting housing	24.7	41.3	8.9	10.8	**1.46 (1.20, 1.79)**	1.11 (0.82, 1.51)	**2.41 (1.82, 3.19)**	**1.83 (1.46, 2.31)**	p = 0.141
Getting medical care	16.1	21.9	4.1	4.9	1.21 (0.95, 1.54)	1.09 (0.71, 1.68)	**2.34 (1.59, 3.45)**	**2.11 (1.54, 2.88)**	p = 0.679
At a store or restaurant	52.6	68.4	8.3	11.9	**1.40 (1.14, 1.70)**	1.22 (0.91, 1.65)	**4.00 (3.03, 5.30)**	**3.51 (2.80, 4.40)**	p = 0.475
Getting credit, loan, mortgage	25.9	42.6	4.1	6.5	**1.46 (1.19, 1.78)**	1.27 (0.85, 1.90)	**3.27 (2.34, 4.56)**	**2.85 (2.11, 3.86)**	p = 0.552
On the street or in a public setting	53.5	69.0	24.4	30.8	**1.39 (1.14, 1.70)**	1.17 (0.96, 1.44)	**2.24 (1.78, 2.82)**	**1.88 (1.60, 2.22)**	p = 0.236
From police or in the courts	31.0	70.3	7.3	17.3	**2.29 (1.87, 2.82)**	**1.63 (1.23, 2.17)**	**3.37 (2.60, 4.35)**	**2.39 (1.88, 3.04)**	p = 0.057

Note: **values in bold** indicate that the 95% CI for the odds ratio excludes 1.0, hence observed difference is **significantly different (p<0.05)**; data presented: observed data (not including missing values).

Correlations (Spearman's *r*) between continuous versions of the explicit and implicit measures of racial discrimination and unfair treatment and additional psychosocial variables (social desirability, racial/ethnic centrality, and hostility) are provided in Table 8, separately for the black and white participants. Among both groups, statistically significant (p<0.05) correlations existed between the EOD, EDS (any), and EDS (race), with these correlations higher among the black compared to white participants: among the black participants, they ranged from 0.388 for EOD with EDS (any) up to 0.673 for EDS (race) with EDS (any). Among the white participants, these correlations ranged only from 0.247 for EDS (race) with EDS (any) up to 0.341 for EOD with EDS (any).

**Table 8:** Correlations between the explicit and implicit racial discrimination measures and additional psychosocial covariates: Spearman's *r* (p-value), 504 black, US-born and 501 white US-born community health center members, *My Body My Story* (Boston, MA, 2008–2010) (observed data).

	Explicit measures			Implicit measures		Additional psychosocial measures		
	EOD	EDS (any)	EDS (race)	IAT: black/white	IAT: me/them	Social desirability	Racial/ethnic centrality	Hostility
**Black participants**								
**EOD**	1.00	–	–	–	–	–	–	–
**EDS (any)**	**0.388 (p<0.0001)** [n = 494]	1.00	–	–	–	–	–	–
**EDS (race)**	**0.438 (p<0.0001)** [n = 494]	**0.673 (p<0.0001)** [n = 495]	1.00	–	–	–	–	–
**IAT: black/white**	0.055 (0.220) [n = 492]	−0.052 (0.255) [n = 485]	0.020 (0.666) [n = 485]	1.00	–	–	–	–
**IAT: me/them**	0.075 (0.098) [n = 491]	0.021 (0.652) [n = 484]	0.061 (0.184) [n = 484]	**0.194 (p<0.0001)** [n = 492]	1.00	–	–	–
**Social desirability**	**−0.173 (0.0001)** [n = 476]	**−0.137 (0.003)** [n = 469]	**−0.132 (0.004)** [n = 469]	−0.044 (0.341) [n = 468]	0.055 (0.238) [n = 466]	1.00	–	–
**Racial/ethnic centrality**	**0.155 (p<0.001)** [n = 502]	0.049 (0.281) [n = 495]	**0.105 (0.020)** [n = 495]	−0.002 (0.969) [n = 493]	0.024 (0.589) [n = 492]	−0.033 (0.470) [n = 477]	1.00	–
**Hostility**	**0.140 (0.002)** [n = 476]	**0.180 (p<0.0001)** [n = 469]	**0.094 (0.042)** [n = 469]	−0.015 (0.752) [n = 468]	−0.018 (0.698) [n = 466]	**−0.314 (p<0.0001)** [n = 477]	0.082 (0.075) [n = 477]	1.00
**White participants**								
**EOD**	1.00	–	–	–	–	–	–	–
**EDS (any)**	**0.341 (p<0.0001)** [n = 496]	1.00	–	–	–	–	–	–
**EDS (race)**	**0.314 (p<0.0001)** [n = 496]	**0.247 (p<0.0001)** [n = 497]	1.00	–	–	–	–	–
**IAT: black/white**	−0.001 (0.983) [n = 490]	0.064 (0.159) [n = 488]	−0.010 (0.819) [n = 488]	1.00	–	–	–	–
**IAT: me/them**	**0.097 (0.031)** [n = 490]	0.005 (0.905) [n = 488]	0.021 (0.644) [n = 488]	−0.072 (0.105) [n = 490]	1.00	–	–	–
**Social desirability**	**−0.169 (p<0.001)** [n = 489]	**−0.251 (p<0.0001)** [n = 487]	**−0.090 (0.047)** [n = 487]	**−0.104 (0.022)** [n = 481]	0.019 (0.685) [n = 481]	1.00	–	–
**Racial/ethnic centrality**	0.076 (0.091) [n = 496]	**0.182 (p<0.0001)** [n = 495]	**0.124 (0.006)** [n = 495]	−0.064 (0.159) [n = 489]	−0.045 (0.324) [n = 489]	**−0.164 (p<0.001)** [n = 488]	1.00	–
**Hostility**	**0.306 (p<0.0001)** [n = 489]	**0.396 (p<0.0001)** [n = 487]	**0.201 (p<0.0001)** [n = 487]	−0.044 (0.335) [n = 481]	−0.067 (0.144) [n = 481]	**−0.317 (p<0.0001)** [n = 490]	**0.142 (0.002)** [n = 488]	1.00

**Note: values in bold** indicate the Spearman correlation coefficient is statistically significant (p<0.05); based on observed data [N], excluding missing values.

As expected, the correlations of the explicit with the implicit measures of racial discrimination were low and not statistically significant, ranging between −0.05 to 0.09. The only exception pertained to the small correlation of EOD with the IAT: me vs them among the white participants (*r* = 0.097, p = 0.031). Additionally, a significant correlation among the two implicit measures occurred only among the black participants (*r* = 0.194; p<0.0001).

Social desirability in turn was, as expected, significantly inversely correlated with the explicit racial discrimination measures among both the black and white participants (i.e., higher social desirability score, lower explicit racial discrimination score), with the *r* among the black participants ranging from −0.173 for EOD to −0.132 for EDS (race), and, among the white participants, from −0.251 for EDS (any) to −0.09 for EDS (race). Conversely, in both racial/ethnic groups, higher hostility was significantly associated with higher self-reports of racial discrimination; these correlations, however, were two times higher among the white compared to the black participants (range of *r*: white: 0.201 to 0.396; black: 0.094 to 0.180). Significant correlations (ranging between 0.105 to 0.182) also occurred between racial/ethnic centrality and several of the explicit racial discrimination measures: among black participants, with the EOD and EDS (race), and among white participants, with the EDS (any) and EDS (race).

By contrast, with only one exception, neither of the IATs in either racial/ethnic group was significantly associated with either social desirability, hostility, or racial/ethnic centrality. Among the white participants, however, a significant albeit small negative correlation existed (r = −0.104; p = 0.022) between social desirability and the IAT: black vs white.

Lastly, Tables 9–11 examine the associations, in univariate and multivariable models, between current smoking and the sociodemographic, economic, the explicit and implicit racial discrimination measures, and psychosocial variables; Table 9 presents results for the EOD, Table 10 for EDS (any), and Table 11 for EDS (race). Variables were included on either *a priori* grounds (age, gender, the explicit and racial discrimination measures, and social desirability) or because they demonstrated significant associations with both the outcome and with race/ethnicity (poverty, education, wealth, and hostility).

**Table 9:** Association of smoking (current smoker vs all others) with explicit measure of racial discrimination (EOD), implicit measures of racial discrimination, and covariates: odds ratio (OR) and 95% confidence interval (CI) for analyses within and comparing the 504 black US-born and 501 white US-born participants, *My Body My Story* (Boston, MA, 2008–2010)(imputed data).

Variables	Black				White				Black-White comparison			
		Multivariable				Multivariable			Multivariable			
	Univariate	Model 1a	Model 1b	Model 1c	Univariate	Model 2a	Model 2b	Model 2c	Model 3a	Model 3b	Model 3c	Model 3d
	OR (95% CI)	OR (95% CI)	OR (95% CI)	OR (95% CI)	OR (95% CI)	OR (95% CI)	OR (95% CI)	OR(95% CI)	OR (95% CI)	OR (95% CI)	OR (95% CI)	OR (95% CI)
***Discrimination: Explicit***												
EOD	1.01 (0.95, 1.08)	1.00 (0.93, 1.08)	–	1.00 (0.93, 1.08)	1.19 (1.07, 1.32)	1.06 (0.94, 1.20)	–	1.06 (0.94, 1.20)	–	–	**1.12 (1.00, 1.25)**	1.06 (0.95, 1.20)
EOD×race/ethnicity	–	–	–	–	–	–	–	–	–	–	0.89 (0.78, 1.01)	0.96 (0.84, 1.10)
***Discrimination: Implicit*** *												
IAT: black vs white as target	0.61 (0.37, 1.01)	–	0.67 (0.38, 1.21)	0.68 (0.38, 1.23)	0.71 (0.46, 1.09)	–	0.74 (0.42, 1.30)	0.72 (0.41, 1.27)	–	–	0.63 (0.39, 1.01)	0.82 (0.49, 1.36)
IAT (Black vs white)×race/ethnicity	–	–	–	–	–	–	–	–	–	–	0.82 (0.41, 1.65)	0.77 (0.37, 1.60)
IAT: me vs them as target	1.21 (0.74, 1.97)	–	1.45 (0.85, 2.47)	1.46 (0.85, 2.50)	1.59 (0.91, 2.79)	–	1.35 (0.71, 2.60)	1.29 (0.67, 2.49)	–	–	1.62 (0.90, 2.93)	1.38 (0.75, 2.57)
IAT (Me vs them)×race/ethnicity	–	–	–	–	–	–	–	–	–	–	0.88 (0.40, 1.90)	1.01 (0.45, 2.26)
***Covariates***												
Race/ethnicity: Black	–	–	–	–	–	–	–	–	**1.50 (1.16, 1.94)**	1.01 (0.76, 1.34)	**2.10 (1.36, 3.24)**	1.17 (0.73, 1.86)
White (referent group)	–	–	–	–	–	–	–	–	1.0	1.0	1.0	1.0
Age (years)	**0.98 (0.96, 1.00)**	**0.98 (0.95, 1.00)**	**0.98 (0.96, 1.00)**	**0.98 (0.95, 1.00)**	**0.97 (0.95, 1.00)**	**0.96 (0.94, 0.99)**	**0.97 (0.94, 0.99)**	**0.97 (0.94, 0.99)**	**0.98 (0.96, 0.99)**	**0.97 (0.95, 0.99)**	**0.98 (0.96, 0.99)**	**0.97 (0.96, 0.99)**
Gender: men vs women (referent group)	**1.57 (1.08, 2.30)**	**1.67 (1.09, 2.56)**	**1.66 (1.10, 2.51)**	**1.71 (1.11, 2.64)**	1.04 (0.71, 1.53)	0.88 (0.56, 1.38)	0.98 (0.62, 1.54)	0.95 (0.59, 1.51)	**1.32 (1.01, 1.73)**	**1.37 (1.03, 1.83)**	1.30 (0.97, 1.74)	1.27 (0.93, 1.74)
women (referent group)	1.0	1.0	1.0	1.0	1.0	1.0	1.0	1.0	1.0	1.0	1.0	1.0
Poverty: <200% poverty	**1.56 (1.07, 2.27)**	1.40 (0.94, 2.08)	1.37 (0.93, 2.04)	1.41 (0.94, 2.10)	1.37 (0.93, 2.01)	0.76 (0.49, 1.19)	0.78 (0.50, 1.22)	0.77 (0.49, 1.22)	–	1.07 (0.80, 1.43)	–	1.07 (0.80, 1.43)
> = 200% poverty (referent group)	1.0	1.0	1.0	1.0	1.0	1.0	1.0	1.0	–	1.0	–	1.0
Education: <HS	**2.65 (1.36, 5.14)**	1.99 (0.95, 4.13)	**2.18 (1.07, 4.46)**	1.95 (0.93, 4.10)	**13.25 (6.25, 28.11)**	**8.75 (3.77, 20.28)**	**7.98 (3.42, 18.61)**	**8.08 (3.46, 18.89)**	–	**4.17 (2.48, 7.00)**	–	**4.14 (2.41, 7.10)**
> = HS and <4 yrs college	**2.35 (1.36, 4.04)**	**1.90 (1.06, 3.41)**	**1.88 (1.05, 3.36)**	**1.87 (1.04, 3.38)**	**5.87 (3.45, 10.01)**	**4.28 (2.37, 7.72)**	**4.18 (2.31, 7.56)**	**4.05 (2.23, 7.36)**	–	**2.92 (1.95, 4.36)**	–	**2.92 (1.93, 4.40)**
> = 4 yrs college (referent group)	1.0	1.0	1.0	1.0	1.0	1.0	1.0	1.0	–	1.0	–	1.0
Wealth: No (<$5000)	**3.52 (1.51, 8.21)**	**3.21 (1.31, 7.88)**	**2.83 (1.16, 6.93)**	**3.05 (1.24, 7.55)**	**5.93 (3.40, 10.33)**	**3.31 (1.74, 6.28)**	**3.24 (1.71, 6.14)**	**3.19 (1.67, 6.08)**	–	**3.39 (2.09, 5.51)**	–	**3.24 (1.97, 5.31)**
Yes (> = $5000) (referent group)	1.0	1.0	1.0	1.0	1.0	1.0	1.0	1.0	–	1.0	–	1.0
Response to unfair treatment: act/quiet	1.35 (0.73, 2.49)	1.12 (0.58, 2.17)	–	1.08 (0.56, 2.09)	1.58 (0.86, 2.89)	1.15 (0.58, 2.31)	–	1.14 (0.56, 2.30)	–	–	1.24 (0.79, v1.94)	1.10 (0.68, 1.76)
accept./talk	1.41 0.85, 2.34)	**1.73 (1.00, 3.00)**	–	1.68 (0.96, 2.92)	0.83 (0.49, 1.41)	0.68 (0.38, 1.23)	–	0.68 (0.37, 1.23)	–	–	1.08 (0.75, 1.56)	1.07 (0.72, 1.58)
accept/quiet	1.34 (0.69, 2.61)	1.34 (0.65, 2.74)	–	1.36 (0.66, 2.81)	1.49 (0.80, 2.78)	1.06 (0.52, 2.18)	–	1.02 (0.50, 2.11)	–	–	1.34 (0.83, 2.16)	1.20 (0.73, 1.97)
act/talk (referent group)	1.0	1.0	–	1.0	1.0	1.0	–	1.0	–	–	1.0	1.0
Social desirability (per 10 units of scale)	**0.94 (0.89, 1.00)**	**0.94 (0.89, 1.00)**	**0.94 (0.89, 1.00)**	**0.94 (0.88, 1.00)**	0.98 (0.92, 1.04)	0.97 (0.90, 1.05)	0.97 (0.89, 1.04)	0.97 (0.90, 1.05)	–	–	0.98 (0.94, 1.03)	**0.95 (0.91, 1.00)**
Hostility	1.03 (0.99, 1.06)	1.01 (0.97, 1.05)	1.01 (0.97, 1.05)	1.01 (0.97, 1.05)	**1.10 (1.06, 1.15)**	**1.06 (1.01, 1.12)**	**1.07 (1.02, 1.12)**	**1.06 (1.01, 1.12)**	–	–	**1.04 (1.01, 1.07)**	**1.03 (1.00, 1.06)**

**Note: values in bold have 95% CI that do not cross 1.00;**

*IAT analyses control for IAT order effects.

**Table 10 pone-0027636-t010:** Association of smoking (current smoker vs all others) with explicit measure of racial discrimination (EDS (any)), implicit measures of racial discrimination, and covariates: odds ratio (OR) and 95% confidence interval (CI) for analyses within and comparing the 504 black US-born and 501 white US-born participants, *My Body My Story* (Boston, MA, 2008–2010)(imputed data).

Variables	Black				White				Black-White comparison			
		Multivariable				Multivariable			Multivariable			
	Univariate	Model 1a	Model 1b	Model 1c	Univariate	Model 2a	Model 2b	Model 2c	Model 3a	Model 3b	Model 3c	Model 3d
	OR (95% CI)	OR (95% CI)	OR (95% CI)	OR (95% CI)	OR (95% CI)	OR (95% CI)	OR (95% CI)	OR(95% CI)	OR (95% CI)	OR (95% CI)	OR (95% CI)	OR (95% CI)
***Discrimination: Explicit***												
EDS (any)	1.00 (0.89, 1.11)	0.94 (0.83, 1.06)	–	0.93 (0.82, 1.05)	**1.15 (1.02, 1.29)**	0.97 (0.84, 1.13)	–	0.98 (0.84, 1.14)	–	–	1.05 (0.93, 1.20)	1.01 (0.88, 1.16)
EDS (any)×race/ethnicity	–	–	–	–	–	–	–	–	–	–	0.89 (0.75, 1.06)	0.94 (0.79, 1.13)
***Discrimination: Implicit*** *												
IAT: black vs white as target	0.61 (0.37, 1.01)	–	0.67 (0.38, 1.21)	0.66 (0.37, 1.20)	0.71 (0.46, 1.09)	–	0.74 (0.42, 1.30)	0.73 (0.42, 1.29)	–	–	0.63 (0.39, 1.00)	0.81 (0.49, 1.36)
IAT (Black vs white)×race/ethnicity	–	–	–	–	–	–	–	–	–	–	0.82 (0.41, 1.63)	0.77 (0.37, 1.59)
IAT: me vs them as target	1.21 (0.74, 1.97)	–	1.45 (0.85, 2.47)	1.48 (0.86, 2.54)	1.59 (0.91, 2.79)	–	1.35 (0.71, 2.60)	1.35 (0.70, 2.60)	–	–	1.72 (0.96, 3.10)	1.45 (0.78, 2.68)
IAT (Me vs them)×race/ethnicity	–	–	–	–	–	–	–	–	–	–	0.83 (0.38, 1.79)	0.99 (0.44, 2.20)
***Covariates***												
Race/ethnicity: Black	–	–	–	–	–	–	–	–	**1.50 (1.16, 1.94)**	1.01 (0.76, 1.34)	**2.38 (1.42, 3.99)**	1.38 (0.79, 2.38)
White (referent group)	–	–	–	–	–	–	–	–	1.0	1.0	1.0	1.0
Age (years)	**0.98 (0.96, 1.00)**	**0.98 (0.95, 1.00)**	**0.98 (0.96, 1.00)**	**0.98 (0.95, 1.00)**	**0.97 (0.95, 1.00)**	**0.96 (0.94, 0.99)**	**0.97 (0.94, 0.99)**	**0.96 (0.94, 0.99)**	**0.98 (0.96, 0.99)**	**0.97 (0.95, 0.99)**	**0.98 (0.96, 0.99)**	**0.97 (0.96, 0.99)**
Gender: men vs women (referent group)	**1.57 (1.08, 2.30)**	**1.71 (1.13, 2.57)**	**1.66 (1.10, 2.51)**	**1.75 (1.16, 2.66)**	1.04 (0.71, 1.53)	0.89 (0.57, 1.40)	0.98 (0.62, 1.54)	0.96 (0.60, 1.53)	**1.32 (1.01, 1.73)**	**1.37 (1.03, 1.83)**	1.31 (0.98, 1.75)	1.32 (0.97, 1.79)
women (referent group)	1.0	1.0	1.0	1.0	1.0	1.0	1.0	1.0	1.0	1.0	1.0	1.0
Poverty: <200% poverty	**1.56 (1.07, 2.27)**	1.43 (0.96, 2.13)	1.37 (0.93, 2.04)	1.44 (0.96, 2.16)	1.37 (0.93, 2.01)	0.76 (0.49, 1.19)	0.78 (0.50, 1.22)	0.78 (0.50, 1.22)	–	1.07 (0.80, 1.43)	–	1.09 (0.81, 1.46)
> = 200% poverty (referent group)	1.0	1.0	1.0	1.0	1.0	1.0	1.0	1.0	–	1.0	–	1.0
Education: <HS	**2.65 (1.36, 5.14)**	1.94 (0.94, 4.00)	**2.18 (1.07, 4.46)**	1.91 (0.92, 3.97)	**13.25 (6.25, 28.11)**	**8.75 (3.77, 20.33)**	**7.98 (3.42, 18.61)**	**8.14 (3.47, 19.09)**	–	**4.17 (2.48, 7.00)**	–	**4.01 (2.34, 6.87)**
> = HS and <4 yrs college	**2.35 (1.36, 4.04)**	**1.90 (1.06, 3.40)**	**1.88 (1.05, 3.36)**	**1.87 (1.04, 3.37)**	**5.87 (3.45, 10.01)**	**4.38 (2.42, 7.93)**	**4.18 (2.31, 7.56)**	**4.16 (2.28, 7.58)**	–	**2.92 (1.95, 4.36)**	–	**2.92 (1.94, 4.41)**
> = 4 yrs college (referent group)	1.0	1.0	1.0	1.0	1.0	1.0	1.0	1.0	–	1.0	–	1.0
Wealth: No (<$5000)	**3.52 (1.51, 8.21)**	**3.13 (1.27, 7.74)**	**2.83 (1.16, 6.93)**	**2.98 (1.20, 7.41)**	**5.93 (3.40, 10.33)**	**3.36 (1.78, 6.37)**	**3.24 (1.71, 6.14)**	**3.23 (1.70, 6.15)**	–	**3.39 (2.09, 5.51)**	–	**3.25 (1.98, 5.33)**
Yes (> = $5000) (referent group)	1.0	1.0	1.0	1.0	1.0	1.0	1.0	1.0	–	1.0	–	1.0
Response to unfair treatment: act/quiet	1.35 (0.73, 2.49)	1.14 (0.59, 2.21)	–	1.10 (0.57, 2.13)	1.58 (0.86, 2.89)	1.17 (0.58, 2.34)	–	1.15 (0.57, 2.33)	–	–	1.27 (0.81, 1.99)	1.10 (0.69, 1.77)
accept./talk	1.41 (0.85, 2.34)	**1.80 (1.03, 3.15)**	–	**1.76 (1.00, 3.08)**	0.83 (0.49, 1.41)	0.69 (0.38, 1.24)	–	0.68 (0.37, 1.23)	–	–	1.10 (0.76, 1.59)	1.08 (0.73, 1.61)
accept/quiet	1.34 (0.69, 2.61)	1.35 (0.66, 2.76)	–	1.37 (0.67, 2.83)	1.49 (0.80, 2.78)	1.08 (0.53, 2.23)	–	1.04 (0.50, 2.15)	–	–	1.34 (0.84, 2.16)	1.20 (0.73, 1.97)
act/talk (referent group)	1.0	1.0	–	1.0	1.0	1.0	–	1.0	–	–	1.0	1.0
Social desirability (per 10 units of scale)	**0.94 (0.89, 1.00)**	**0.94 (0.88, 1.00)**	**0.94 (0.89, 1.00)**	**0.94 (0.88, 0.99)**	0.98 (0.92, 1.04)	0.97 (0.89, 1.05)	0.97 (0.89, 1.04)	0.97 (0.89, 1.05)	–	–	0.98 (0.94, 1.03)	**0.95 (0.90, 1.00)**
Hostility	1.03 (0.99, 1.06)	1.01 (0.97, 1.06)	1.01 (0.97, 1.05)	1.02 (0.98, 1.06)	**1.10 (1.06, 1.15)**	**1.07 (1.02, 1.13)**	**1.07 (1.02, 1.12)**	**1.07 (1.02, 1.13)**	–	–	**1.05 (1.02, 1.08)**	**1.03 (1.00, 1.07)**

**Note: values in bold have 95% CI that do not cross 1.00;**

*IAT analyses control for IAT order effects.

**Table 11:** Association of smoking (current smoker vs all others) with explicit measure of racial discrimination (EDS (race)), implicit measures of racial discrimination, and covariates: odds ratio (OR) and 95% confidence interval (CI) for analyses within and comparing the 504 black US-born and 501 white US-born participants, *My Body My Story* (Boston, MA, 2008–2010)(imputed data).

Variables	Black				White				Black-White comparison			
		Multivariable				Multivariable			Multivariable			
	Univariate	Model 1a	Model 1b	Model 1c	Univariate	Model 2a	Model 2b	Model 2c	Model 3a	Model 3b	Model 3c	Model 3d
	OR (95% CI)	OR (95% CI)	OR (95% CI)	OR (95% CI)	OR (95% CI)	OR (95% CI)	OR (95% CI)	OR(95% CI)	OR (95% CI)	OR (95% CI)	OR (95% CI)	OR (95% CI)
***Discrimination: Explicit***												
EDS (race)	1.04 (0.94, 1.14)	1.04 (0.93, 1.15)	–	1.03 (0.93, 1.15)	**1.16 (1.01, 1.34)**	1.09 (0.93, 1.29)	–	1.11 (0.94, 1.31)	–	–	1.10 (0.95, 1.27)	1.10 (0.94, 1.28)
EDS (any)×race/ethnicity	–	–	–	–	–	–	–	–	–	–	0.92 (0.77, 1.09)	0.96 (0.80, 1.16)
***Discrimination: Implicit*** *												
IAT: black vs white as target	0.61 (0.37, 1.01)	–	0.67 (0.38, 1.21)	0.68 (0.38, 1.24)	0.71 (0.46, 1.09)	–	0.74 (0.42, 1.30)	0.73 (0.41, 1.28)	–	–	0.64 (0.40, 1.02)	0.83 (0.50, 1.38)
IAT (Black vs white)×race/ethnicity	–	–	–	–	–	–	–	–	–	–	0.81 (0.41, 1.62)	0.77 (0.37, 1.60)
IAT: me vs them as target	1.21 (0.74, 1.97)	–	1.45 (0.85, 2.47)	1.44 (0.84, 2.47)	1.59 (0.91, 2.79)	–	1.35 (0.71, 2.60)	1.33 (0.69, 2.56)	–	–	1.71 (0.95, 3.07)	1.43 (0.78, 2.65)
IAT (Me vs them)×race/ethnicity	–	–	–	–	–	–	–	–	–	–	0.83 (0.38, 1.79)	0.97 (0.44, 2.16)
***Covariates***												
Race/ethnicity: Black	–	–	–	–	–	–	–	–	**1.50 (1.16, 1.94)**	1.01 (0.76, 1.34)	**1.87 (1.28, 2.72)**	1.12 (0.75, 1.68)
White (referent group)	–	–	–	–	–	–	–	–	1.0	1.0	1.0	1.0
Age (years)	**0.98 (0.96, 1.00)**	**0.98 (0.95, 1.00)**	**0.98 (0.96, 1.00)**	**0.98 (0.95, 1.00)**	**0.97 (0.95, 1.00)**	**0.96 (0.94, 0.99)**	**0.97 (0.94, 0.99)**	**0.96 (0.94, 0.99)**	**0.98 (0.96, 0.99)**	**0.97 (0.95, 0.99)**	**0.98 (0.96, 0.99)**	**0.97 (0.96, 0.99)**
Gender: men vs women (referent group)	**1.57 (1.08, 2.30)**	**1.63 (1.08, 2.47)**	**1.66 (1.10, 2.51)**	**1.68 (1.11, 2.54)**	1.04 (0.71, 1.53)	0.88 (0.56, 1.38)	0.98 (0.62, 1.54)	0.95 (0.59, 1.51)	**1.32 (1.01, 1.73)**	**1.37 (1.03, 1.83)**	1.29 (0.97, 1.72)	1.28 (0.95, 1.74)
women (referent group)	1.0	1.0	1.0	1.0	1.0	1.0	1.0	1.0	1.0	1.0	1.0	1.0
Poverty: <200% poverty	**1.56 (1.07, 2.27)**	1.39 (0.94, 2.07)	1.37 (0.93, 2.04)	1.40 (0.94, 2.08)	1.37 (0.93, 2.01)	0.76 (0.49, 1.19)	0.78 (0.50, 1.22)	0.78 (0.49, 1.22)	–	1.07 (0.80, 1.43)	–	1.07 (0.80, 1.44)
> = 200% poverty (referent group)	1.0	1.0	1.0	1.0	1.0	1.0	1.0	1.0	–	1.0	–	1.0
Education: <HS	**2.65 (1.36, 5.14)**	**2.06 (1.00, 4.28)**	**2.18 (1.07, 4.46)**	2.02 (0.96, 4.23)	**13.25 (6.25, 28.11)**	**8.92 (3.83, 20.77)**	**7.98 (3.42, 18.61)**	**8.33 (3.54, 19.58)**	–	**4.17 (2.48, 7.00)**	–	**4.22 (2.46, 7.24)**
> = HS and <4 yrs college	**2.35 (1.36, 4.04)**	**1.94 (1.08, 3.47)**	**1.88 (1.05, 3.36)**	**1.90 (1.05, 3.43)**	**5.87 (3.45, 10.01)**	**4.44 (2.45, 8.05)**	**4.18 (2.31, 7.56)**	**4.26 (2.33, 7.77)**	–	**2.92 (1.95, 4.36)**	–	**2.99 (1.98, 4.52)**
> = 4 yrs college (referent group)	1.0	1.0	1.0	1.0	1.0	1.0	1.0	1.0	–	1.0	–	1.0
Wealth: No (<$5000)	**3.52 (1.51, 8.21)**	**3.29 (1.34, 8.09)**	**2.83 (1.16, 6.93)**	**3.13 (1.26, 7.73)**	**5.93 (3.40, 10.33)**	**3.29 (1.73, 6.26)**	**3.24 (1.71, 6.14)**	**3.15 (1.65, 6.02)**	–	**3.39 (2.09, 5.51)**	–	**3.26 (1.99, 5.36)**
Yes (> = $5000) (referent group)	1.0	1.0	1.0	1.0	1.0	1.0	1.0	1.0	–	1.0	–	1.0
Response to unfair treatment: act/quiet	1.35 (0.73, 2.49)	1.12 (0.58, 2.17)	–	1.08 (0.56, 2.09)	1.58 (0.86, 2.89)	1.13 (0.56, 2.27)	–	1.11 (0.55, 2.25)	–	–	1.24 (0.79, 1.95)	1.08 (0.67, 1.73)
accept./talk	1.41 (0.85, 2.34)	1.72 (0.99, 2.98)	–	1.66 (0.95, 2.90)	0.83 (0.49, 1.41)	0.68 (0.38, 1.24)	–	0.68 (0.37, 1.23)	–	–	1.08 (0.75, 1.56)	1.06 (0.72, 1.57)
accept/quiet	1.34 (0.69, 2.61)	1.34 (0.66, 2.75)	–	1.36 (0.66, 2.82)	1.49 (0.80, 2.78)	1.06 (0.51, 2.17)	–	1.01 (0.49, 2.09)	–	–	1.35 (0.84, 2.16)	1.19 (0.72, 1.95)
act/talk (referent group)	1.0	1.0	–	1.0	1.0	1.0	–	1.0	–	–	1.0	1.0
Social desirability (per 10 units of scale)	**0.94 (0.89, 1.00)**	**0.94 (0.89, 1.00)**	**0.94 (0.89, 1.00)**	**0.94 (0.89, 1.00)**	0.98 (0.92, 1.04)	0.97 (0.90, 1.05)	0.97 (0.89, 1.04)	0.97 (0.90, 1.05)	–	–	0.98 (0.94, 1.03)	**0.95 (0.91, 1.00)**
Hostility	1.03 (0.99, 1.06)	1.01 (0.97, 1.05)	1.01 (0.97, 1.05)	1.01 (0.97, 1.05)	**1.10 (1.06, 1.15)**	**1.06 (1.01, 1.12)**	**1.07 (1.02, 1.12)**	**1.06 (1.01, 1.12)**	–	–	**1.05 (1.01, 1.08)**	**1.03 (1.00, 1.06)**

Note: values in bold have 95% CI that do not cross 1.00;

*IAT analyses control for IAT order effects.

Among the black participants, none of the explicit or implicit measures of racial discrimination were associated with being a current smoker, whether in univariate analyses or in analyses that controlled for sociodemographic and psychosocial variables (Models 1a–1c). Instead, the two strongest and statistically significant variables associated with current smoking were: (1) a lack of wealth (odds ratios (ORs) in the adjusted models ranging from 2.83 (95% CI 1.16, 6.93) to 3.29 (95% CI 1.34, 8.09, depending on model covariates), and (2) lower education (ORs ranging between slightly under 2 to slightly over 3 for less than high school and also high school to less than 4 years college versus 4+ years of college). Social desirability and age also were consistently modestly inversely associated with the odds of current smoking (OR (per 10 units of the scale) = 0.94 (95% CI 0.89, 1.00) and 0.98 (95% CI 0.95, 10.00) respectively); men were more likely to be current smokers than women.

By contrast, among the white participants (Models 2a–2c), both the EOD and EDS (any), in the univariate analyses only, were modestly but significantly associated with cigarette smoking (ORs on the order of 1.2). Second, the ORs for lower education were greater (approximately 8 for less than high school and 4 for at least high school but less than 4 years college, as compared to 4 or more years of college). Third, whereas no association existed between social desirability and smoking, or gender and smoking, a positive significant albeit modest association did exist for hostility (OR on the order of 1.1).

Finally, in a model adjusting for only age and gender (Model 3a), the black versus white odds for being a current smoker were 1.50 (95% 1.16, 1.94). Additionally adjusting for the socioeconomic measures (Model 3b) rendered this difference null (OR = 1.01, 95% CI 0.76, 1.34). By contrast, additionally adjusting for the explicit and implicit racial discrimination (including their interactions with race/ethnicity) and the psychosocial variables but not for the socioeconomic variables (Model 3c) increased the black vs white odds ratio: to 2.10 (95% CI 1.36, 3.24) for the model that included the EOD, to 2.38 (95% CI 1.42, 3.99) for the model that included EDS (any), and to 1.87 (95% CI 1.28, 2.72) for the model that included EDS (race). In these models, the only explicit discrimination measure that was significantly associated with being a current smoker was the EOD (OR = 1.12 (95% CI 1.00, 1.25); interaction of EOD×race/ethnicity: OR = 0.89 (95% CI 0.78, 1.01), indicating the impact was less among the black compared to white participants); the IAT: me vs them also tended to be associated (OR on the order of 1.7 in all 3 of the explicit discrimination models, with the IAT×race/ethnicity interaction term non-significant in all models, and its OR between 0.8 and 0.9). Finally, in a model adjusting simultaneously for the sociodemographic, socioeconomic, and discrimination measures (Model 3d), the black vs white odds was again rendered statistically non-significant, and lower education, lack of wealth, younger age, lower social desirability, and higher hostility all remained significantly associated with being a current smoker.

## Discussion

Our investigation, the first jointly to use implicit and explicit measures of racial discrimination in a large community-based study, provides clear evidence that the implicit and explicit measures are, as expected, not equivalent. Of direct relevance to research on racial discrimination and health, the results additionally underscore that studies employing solely explicit self-report data on racial discrimination are incomplete if they fail to take into account issues of social desirability, as is the case with the preponderance of research on racial discrimination and health [1]–[6]; also problematic is the common practice of treating responses across racial/ethnic groups as equivalent. A secondary finding, replicating that of other recent research [25], [64], [65], is that it is also inappropriate and problematic to treat explicit self-report measures of racial discrimination and unfair treatment (without attribution) as equivalent, as has also occurred in the public health literature [77]–[80], because they are conceptually and empirically dissociated. Also essential is appropriately characterizing study participants' socioeconomic position, in light of not only persistent racial/ethnic inequities in resources but also differential associations of diverse measures of socioeconomic position, within and across racial/ethnic groups, with both the explicit self-reports of racial/ethnic discrimination, and also the psychosocial variables and selected health outcome.

### Study limitations

Several caveats must be considered, however, before offering an interpretation of our findings. First, our study was cross-sectional, limiting causal inference, even as we did distinguish between childhood and adult worries about racial discrimination and exposure to socioeconomic deprivation, and also between lifetime and recent self-reports of racial discrimination. Second, our study population was deliberately restricted, for the methodologic and substantive reasons described above, to US-born English-speaking self-identified black and white adult members of four community health centers in one large US northeastern urban city (Boston, MA). Their socioeconomic profile, however, resembles that of Boston and US black and white working class and middle- to low-income adults [81], [82], who comprise the majority of both populations, and among whom racial/ethnic disparities in economic resources at each socioeconomic level are well-documented [81], [82]. Thus, our study results are likely salient for research on racial discrimination and the health of US-born black Americans, including in comparison to US-born white Americans, even though they cannot be generalized to other US racial/ethnic groups, to immigrant and to non-English speaking populations, to much more highly educated and more affluent populations, or to populations residing in other urban or rural regions in the US. Nevertheless, the concerns we raise about measurement issues for exposure to racial discrimination are likely to be relevant to health research on racial discrimination in any population and country context. Third, in these analyses we examined only one health outcome (cigarette smoking), since our main emphasis was on ascertaining the patterns of association between the explicit and implicit measures of racial discrimination, both with each other and also key socioeconomic and psychosocial covariates. In future papers we will analyze a range of health outcomes, informed by the results of this investigation.

### Study findings: context and interpretation

First, regarding the implicit measures, we note that the magnitude of the observed IAT effects is on par with those detected for other more widely used IAT measures [30]–[32], thereby placing results within a credible range of effect estimates. Moreover, the magnitude of the IAT effect we observed for the college educated black participants in *My Body, My Story* was similar to that which we observed in the highly educated sample of 442 self-identified black participants who participated in our prior web-based study that used these same two IAT measures [9]; also virtually identical for both studies was the magnitude of the association between the two IAT measures and also of each with the EOD [9]. With regard to the results for the white population, and also black/white comparisons, the only other comparable published data are from our prior small pilot study (n = 31; 13 white, 18 black) [8], for which we found the average effect size for the IAT: black vs white was higher for its white participants (mean = 0.29 (SD = 0.36)) as compared to that observed in the current study, even as the effect estimates for the IAT: me vs them were similar; results for the black participants were similar. Given the small sample size, however, no real comparison of effect sizes across these two studies can be meaningfully offered.

Keeping in mind the limited empirical data available for comparison, what nevertheless stands out, pending replication in future studies, are three key findings:

1. the significantly higher IAT effects for the black as compared to white participants for both IAT measures (IAT: black vs white and IAT: me vs them);
2. the significant correlation among the black participants only between the two IAT measures; and
3. the low non-significant association of the implicit discrimination measures with: (a) the explicit measures of racial discrimination, and (b) the other psychosocial variables (social desirability, racial/ethnic centrality, and hostility); the only two exceptions, both occurring among solely the white participants were: the weak positive association with the EOD measure and the weak negative association with the IAT: black vs white.

Together, these results suggest that, as expected, the implicit discrimination measures: (a) are generally immune to self-representation and self-identity [8], [9], [30]–[33]; and (b) reveal the black participants are more likely to associate both themselves and their racial/ethnic group with being a target of discrimination than the white participants. Also plausible is the finding that significant correlations existed between the two IATs (for group and self as targets of discrimination) only among the black participants, given their belonging to a group historically defined in part by being subjected to racial discrimination. Further lending support to our results are findings of other research documenting low to medium correlations between implicit and explicit measures for phenomena subject to self-representational bias (e.g., racial discrimination) [30]–[33], [66]. A 2005 meta-analysis of correlations between IATs and explicit self-report measures, for example, found that although the on-average correlation was 0.24, the 90% credible interval ranged from 0.11 to 0.47 [83].

That said, the detection of small yet statistically significant associations (at p<0.05), among whites only, of: (i) the IAT: me vs them with the EOD (higher IAT effect with higher EOD score), but not the two other explicit discrimination measures, and (ii) the IAT: black vs white with social desirability (with higher IAT effect associated with lower social desirability score), are findings that would need to be replicated, to rule out chance (e.g., due to multiple comparisons). For example, had we *a priori* set the p-value for significant associations to p<0.01, rather than p<0.05, given multiple comparisons, neither of these associations would have been deemed statistically significant, whereas the association between the two IAT measures among the black participants would have remained statistically significant (since its p-value was <0.0001).

Second, our findings for the explicit measures of discrimination underscore the need to consider how these self-report data need to be interpreted in relation to issues of social and economic power, resources, and identity. As our findings for social desirability suggest – including not only its high levels among the black participants, regardless of socioeconomic position, but also its marked inverse socioeconomic gradient among the white participants (highest among the white women with the least resources; lowest among the white men with the most resources) – two phenomena likely are at issue. One pertains to the conscious attributions people make for reasons for adverse experiences they encounter, which likely are shaped by their understanding of their societal context [1], [2], [10]–[12], [14]–[17], [69]. The second concerns their likelihood of explicitly reporting these conscious attributions, which hinges on the extent to which their responses are muted by concerns about social desirability [1], [8], [9].

Of note, in the prior 14 studies on racial discrimination and health that included diverse measures of social desirability [9], [17]–[29], only 3 reported on the association between their selected measure and the explicit measures of discrimination. Of these, one based on a sample of working class participants in the Boston area found no association between social desirability and the EOD, but did find evidence of a slight positive association with the EDS [18]; a second, based on a small sample of 49 African American men reported no association between social desirability and the EDS, but the confidence intervals were wide [27]; and the third, based on a sample of Turkish and Moroccan adolescents in The Netherlands, reported no association between social desirability and their self-report measures of racial discrimination [26]. The paucity of comparisons, along with the likely importance of addressing issues of social desirability in research on racial discrimination and health (especially given the high levels in the black compared to white participants), suggests further research on this issue is warranted.

Further aiding with interpretation of the explicit self-report discrimination measures are our findings that their psychosocial correlates may differ by race/ethnicity. Of particular note are our observations of: (a) much stronger associations between hostility and self-reported experiences of racial discrimination among the white compared to black participants; (b) associations of the racial/ethnic centrality score with only the EOD and EDS (race) among the black participants, and principally the EDS (any) but also EDS (race) among the white participants; and (c) an association between racial/ethnic centrality and hostility among only the white participants. To our knowledge, these empirical patterns of associations have not been reported previously. Taken together, they lend support to the hypothesis that self-reports of racial discrimination among historically racially dominant versus subordinated groups reflect different expectations, with the former potentially more linked to resentments about loss of privileges associated with charges of “reverse discrimination” [10]–[12].

Also noteworthy are the gender differences in the domains of discrimination reported by the black participants, a finding that points to: (a) the importance of analyzing gendered racism [1], [2], [10]–[12], [46] and (b) from both an etiologic and intervention perspective, ensuring that self-report measures capture the domains in which discrimination is reported, as opposed to solely people's summary appraisal of feelings and frequencies without reference to the situations involved, as is the case for several extant measures [1], [3], [4]–[6].

Lastly, with regard to current smoking and racial discrimination, we note that the main reason for including a health-related outcome in this first set of analyses was to underscore the public health salience of refining methods for analyzing the health impact of racial discrimination [84]. Contributing to our choice of outcome, among the 12 studies published as of July 2011 that analyzed associations between smoking and self-reported exposure to racial discrimination which included US black or both black and white adults, 8 reported positive associations [85]–[91]; notably, all but one [88] reported prevalences of current smoking at half the high levels observed in our study. Four studies, however, like ours, reported no association [18], [92]–[94]. Keeping in mind the high rates of smoking in our population (which constrains the variability to be explained), our findings of: (1) strong associations between lower education and current smoking, one reported in many studies [95], combined with (2) large differences in educational level and economic resources among the black compared to white participants, resulted in (3) control for socioeconomic position rendering null the observed excess black risk for being a current smoker. By contrast, controlling for the discrimination measures in models that did not include the socioeconomic measures increased this risk (especially for EDS (any)). The most plausible interpretation involves four patterns observed in our study population: (a) the black participants were far more likely to experience economic deprivation than the white participants; (b) the direction of the socioeconomic gradient for self-reported experiences of racial discrimination went in opposite directions for the black versus white participants (i.e., positive versus inverse, respectively); (c) the inverse socioeconomic gradient for smoking was stronger among the white compared to black participants; and (d) among white participants only, in univariate analyses (not adjusted for socioeconomic position), there was a slight positive association between self-reported experiences of racial discrimination and risk of smoking. Together, these patterns of association would account for both: (1) the residual confounding that elevated the risk of smoking among the black compared to white participants in models that adjusted for racial discrimination and other psychosocial covariates without also controlling for socioeconomic position, and (2) the elimination of this excess risk in models that additionally controlled for socioeconomic position. The larger implication is that analyzing the health consequences of racial discrimination on health requires not only implicit and explicit measures of racial discrimination but also consideration of socioeconomic position, itself linked to racial discrimination both historically and in the present [1]–[3], [36], [45]–[47].

### Implications for future research on racial discrimination and health

In summary, our study provides evidence, among a population-based sample of self-identified black and white US-born members of community health centers, of stark racial/ethnic inequities in economic resources and exposure to racial discrimination, with the black participants more likely than the white participants to be impoverished, to make stronger associations between themselves and their group as a target of racial discrimination, to self-report exposure to racial discrimination, and to manifest higher social desirability scores. Exposing these patterns of racial discrimination requires frameworks and methods attuned to how issues of societal power and inequity not only drive the phenomenon under study, i.e., racial discrimination, but can also affect the measurement of exposure and its effects. The point is not whether implicit versus explicit measures of racial discrimination are “better”; rather, our evidence suggests each provides important non-equivalent information about exposure – and that neither can be analyzed without regard for societal, including economic, context [84]. As guided by the ecosocial construct of embodiment [34]–[36], [84], the goal is to triangulate evidence, whereby studies can be enriched by including data on what people self-report, what implicit associations they make, and what their bodies recount. Hence our study's name – *My Body, My Story* – because to understand people's health, and the causes of health inequities, it matters what we can say, what we are unable to say, and what our bodies say [1], [35], [36], [84].

In future studies, we will report on the salience of the implicit and explicit measures of racial discrimination for outcomes pertaining to chronic disease risk among the *My Body, My Story* participants. In the interim, we believe our results provide support for the suggestion that future research on racial discrimination and the health – whether conducted among US-born black and white Americans, among additional US racial/ethnic and immigrant groups, or in other country contexts – should consider empirically testing the utility of employing both implicit and explicit measures of racial discrimination, in conjunction with appropriate data on socioeconomic resources and social desirability.
